# YTHDF2 inhibition potentiates radiotherapy anti-tumor efficacy

**DOI:** 10.1016/j.ccell.2023.04.019

**Published:** 2023-05-25

**Authors:** Liangliang Wang, Xiaoyang Dou, Shijie Chen, Xianbin Yu, Xiaona Huang, Linda Zhang, Yantao Chen, Jiaai Wang, Kaiting Yang, Jason Bugno, Sean Pitroda, Xingchen Ding, Andras Piffko, Wei Si, Chao Chen, Hualiang Jiang, Bing Zhou, Steven J. Chmura, Cheng Luo, Hua Laura Liang, Chuan He, Ralph R Weichselbaum

**Affiliations:** 1. Department of Radiation and Cellular Oncology, University of Chicago, Chicago IL 60637 USA; 2. Ludwig Center for Metastasis Research, University of Chicago, Chicago, IL 60637 USA; 3. Department of Chemistry, Department of Biochemistry and Molecular Biology, and Institute for Biophysical Dynamics, The University of Chicago, Chicago, IL 60637, USA; 4. Howard Hughes Medical Institute, University of Chicago, Chicago, IL 60637, USA; 5. State Key Laboratory of Drug Research, Shanghai Institute of Materia Medica, Chinese Academy of Sciences, Shanghai 201203, China; 6. The Committee on Clinical Pharmacology and Pharmacogenomics, University of Chicago, Chicago, IL 600637, USA; 7. Shandong Cancer Hospital and Institute, Shandong First Medical University and Shandong Academy of Medical Sciences, Jinan 250117, China; 8. Department of Neurosurgery, University Medical Center Hamburg-Eppendorf, Hamburg 20246, Germany; 9. State Key Laboratory of Animal Nutrition, Institute of Animal Science, Chinese Academy of Agricultural Sciences, Beijing 100193, China; 10. Zhongshan Institute for Drug Discovery, Shanghai Institute of Materia Medica, Chinese Academy of Sciences, Zhongshan 528437, China; 11. Department of Biochemistry and Molecular Biology, University of Chicago, Chicago, IL 60637, USA; 12. These authors contributed equally: Liangliang Wang, Xiaoyang Dou, Shijie Chen; 13. Lead contact

## Abstract

RNA *N*^6^-methyladenosine (m^6^A) modification is implicated in cancer progression. However, the impact of m^6^A on the anti-tumor effects of radiotherapy and the related mechanisms are unknown. Here we show that ionizing radiation (IR) induces immunosuppressive myeloid-derived suppressor cell (MDSC) expansion and YTHDF2 expression in both murine models and humans. Following IR, loss of *Ythdf2* in myeloid cells augments antitumor immunity and overcomes tumor radioresistance by altering MDSC differentiation, and inhibiting MDSC infiltration and suppressive function. The remodeling of the landscape of MDSC populations by local IR is reversed by *Ythdf2* deficiency. IR-induced YTHDF2 expression relies on NF-κB signaling; YTHDF2 in turn leads to NF-κB activation by directly binding and degrading transcripts encoding negative regulators of NF-κB signaling, resulting in an IR-YTHDF2-NF-κB circuit. Pharmacological inhibition of YTHDF2 overcomes MDSC-induced immunosuppression and improves combined IR and/or anti-PD-L1 treatment. Thus, YTHDF2 is a promising target to improve radiotherapy (RT) and RT/immunotherapy combinations.

## aTOC

Wang et al. discover that loss of *Ythdf2* in myeloid cells augments antitumor immunity of ionizing radiation (IR). The IR-YTHDF2-NF-κB circuit governs MDSC migration and suppressive function. YTHDF2 inhibition enhances efficacy of radiation and checkpoint inhibitor.

## Graphical Abstract

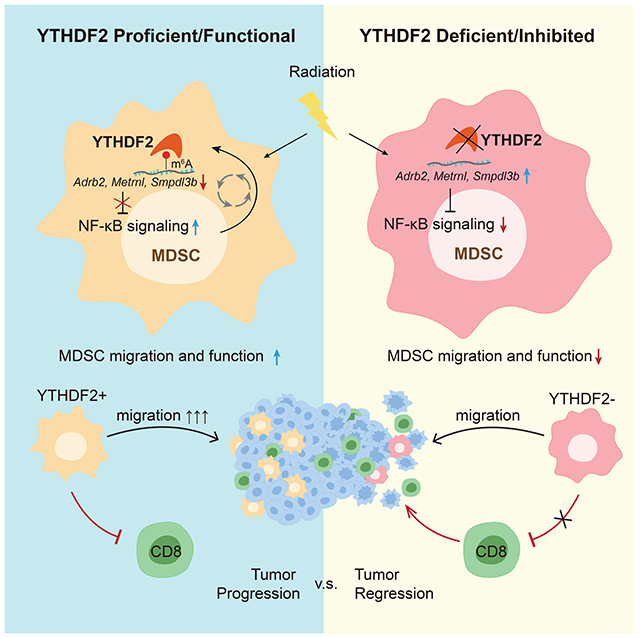

## Introduction

Radiation therapy (RT) is employed in 50-60% of cancer patients.^1,2^ Despite continuous technological and therapeutic improvements, the majority of patients experiences treatment failure either locally (due to tumor radioresistance) or at distant metastatic sites. Tumor cell radioresistance contributes to treatment failure and radiosensitizing therapeutic strategies have generally focused on inhibiting DNA repair or increasing DNA damage.^3^ The immune contexture is vital in radiocurability.^4^ Preclinical data indicates an immune stimulatory effect of ionizing radiation (IR) alone or in combination with checkpoint blockade.^5–9^ Encouraged by these promising preclinical results, an increasing number of clinical trials combining checkpoint blockade with IR has been launched (reviewed in ^10,11^). Despite encouraging individual patient responses, only two randomized trials have shown positive results in terms of improving survival.^12,13^ These trials utilized checkpoint blockade after chemo/radiotherapy to inhibit microscopic metastatic disease, however a consistent meaningful interaction between checkpoint immunotherapy and radiotherapy in humans has yet to be established.^14^

Myeloid-derived suppressor cells (MDSCs) have emerged as crucial negative regulators of the antitumor immune response. Inhibiting immunosuppressive effects of cancer therapy is a pivotal step towards therapeutic success.^15^ In the setting of cancer, MDSCs maintain cellular plasticity and can be reprogrammed into various myeloid cells depending on the tumor, normal tissue microenvironments as well as various treatments.^16,17^ Mechanisms of immune suppression by MDSCs include secretion or expression of immunoregulatory factors,^18,19^ which downregulate cytotoxic CD8^+^ T cell function. Tumor infiltration of MDSCs may account for preclinical/clinical radio- and immune checkpoint blockade resistance.^19–21^ In pre-clinical studies, both fractionated and hypo-fractionated treatment regimens resulted in MDSC expansion,^22–25^ which lead to tumor radioresistance^24,26,27^. Clinical studies also showed that radiotherapy induces MDSCs expansion,^24,25,28,29^ immunosuppression,^30^ and these effects appeared to associate with adverse patient outcomes.^25,31–33^ There are several ongoing or recently completed phase 1/2 clinical trials aiming to modulate MDSCs to enhance immunotherapy in distinct ways.^19,34^ Examples include MDSC depletion,^35^ MDSC migration blockade (20 trials using CCR5/CCR2 antagonists and 52 trials using CSFR antagonists),^36^ MDSC function blockage to attenuate their suppressive effects (targeting phosphodiesterase 5 to reduce iNOS and ARG1 production),^37^ and induction of MDSC differentiation by utilizing all-trans-retinoic acid (ATRA).^38^ Despite these studies, no randomized trials have shown improved survival.

*N*^6^-methyladenosine (m^6^A), the most prevalent eukaryotic mRNA modification, regulates the stability and translation of modified mRNAs.^39,40^ m^6^A is dynamically regulated by “writers” (methyltransferase complex: METTL3, METTL14, and WTAP) that install m^6^A methylation; “erasers” (demethylases: FTO and ALKBH5) that remove m^6^A marks; and “readers” (YTHDF1/2/3 and YTHDC1/2) that recognize m^6^A-modified RNA to regulate RNA metabolisms.^41^ Among the m^6^A reader proteins, YTHDF1 facilitates mRNA translation; YTHDF2 promotes mRNA degradation; and YTHDF3 promotes both translation and RNA degradation depending on the biological context.^42,44^ Recent studies have suggested m^6^A readers and erasers are implicated in tumor growth in various cancer types.^45–49^ Only few studies have reached the impact of reader proteins on antitumor immune response: loss of *Ythdf1* enhanced the cross-priming activity via decreasing lysosomal proteases in classical dendritic cells and suppressed tumor growth.^50^ YTHDF2 affects many biological processes including cell cycle progression,^51^ response to stress,^52^ and regulation of hematopoietic stem cell expansion.^47,53–55^ However, the intrinsic role of YTHDF2 in immune cells especially in relation to radiotherapy and immunotherapy has not been explored in depth.

Here we report that, in a clinical trial, IR mediated an increase of YTHDF2-expressing MDSC population following radiotherapy (RT), which associated with metastasis progression post-RT. In murine models, loss of *Ythdf2* in myeloid cells augmented the efficacy of local tumor IR by altering MDSC differentiation, inhibiting MDSC trafficking into tumors and attenuating their suppressive functions. The induction of YTHDF2 by IR via NF-κB activation resulted in downregulation of its direct targets *Adrb2*, *Metrnl* and *Smpdl3b*, which negatively regulate NF-κB signaling. The YTHDF2 inhibitor DC-Y13-27 identified through a small molecule drug screen enhanced the antitumor effects of radiotherapy and radio-immunotherapy combinations in a manner similar to the deletion of YTHDF2. The alleviation of immunosuppression through YTHDF2 blockade is a therapeutic paradigm that not only improves local tumor control but also suppresses distant metastasis.

## Results

### Local tumor irradiation increases tumor-associated myeloid cells expressing YTHDF2

To investigate the cellular and molecular contexture of the tumor immune microenvironment (TME) following high dose radiation used in ablative radiotherapy, we characterized CD45^+^ immune cells isolated from irradiated (4 days after 20 Gy-IR) and non-irradiated MC38 tumors by high-throughput single-cell RNA sequencing (scRNA-seq). We identified five major cell lineages including T cells, natural killer (NK) cells, dendritic cells (DCs), monocytes, and macrophages, based on gene expression signatures (Figure S1A). We then characterized the changes of these cell subtypes in irradiated tumors compared with non-irradiated tumors. The proportion of T cells was slightly decreased, while an NK subset (*Klrb1c*_NK, C04), two DC subsets (*Ccl22*_cDC1, C11 and *Cd209a*_cDC2, C10), distinct subsets of macrophages and monocytes (C03 and C05), and neutrophil subsets (C12) were increased post-IR (Figures S1B–S1D). Of note, C03 cells showed upregulated *Vegfa* expression and C05 cells showed upregulated *Nr4a1* expression, suggesting their tendency towards the M2 phenotype. Thus, IR markedly changes the TME in ways that alter tumor-infiltrating immune cells including NKs, DCs, macrophages and neutrophils.

**Figure 1.**
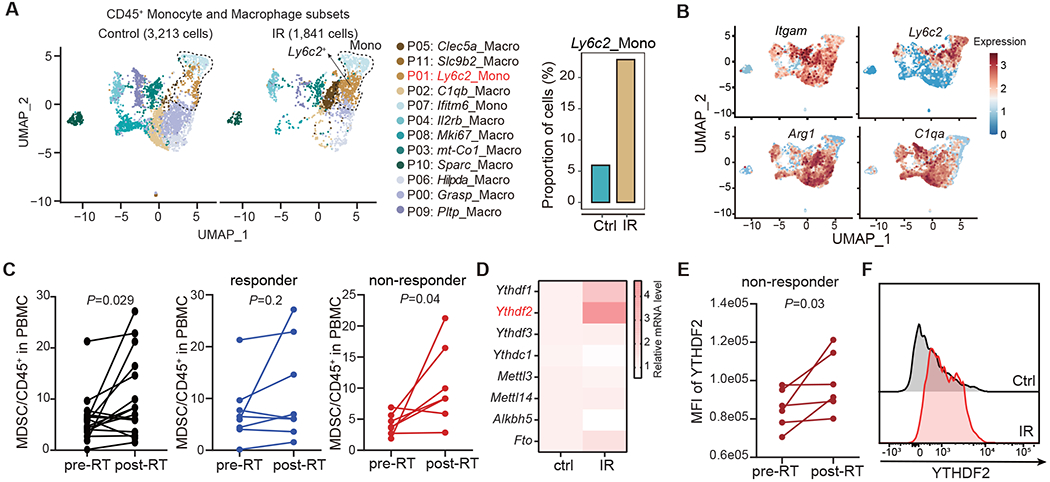
Local tumor irradiation increases tumor-associated myeloid cells expressing YTHDF2. **(A)** UMAP plot of scRNA-seq data showing the different myeloid cell clusters of CD45^+^ immune cells, which were isolated from non-irradiated (Control) and irradiated (IR) MC38 mouse tumors, respectively (left). Bar plot showing the proportion of ‘P01: *Ly6c2*_Mono’ cluster in control and irradiated tumors, respectively (right). CD45^+^ immune cells were obtained from four pooled MC38 tumor-bearing mice four days after IR. **(B)** Expression levels of selected genes identifying ‘P01:*Ly6c2*_Mono’ cluster as MDSC in UMAP space. **(C)** Flow cytometry analysis of MDSCs in PBMCs from cancer patients with lung metastasis (pre-RT vs. post-RT). The post-RT blood samples were collected approximately 1-3 weeks (median 14 days) after the pre-RT samples. **(D)** Heatmap showing the mRNA expression of m^6^A-related genes (identified by qPCR analysis) in MDSCs from non-irradiated and irradiated MC38 tumors three days after IR. One representative result (of three independent experiments) with three technical replicates was shown. **(E)** Mean Fluorescent Intensity (MFI) of YTHDF2 in MDSCs of PBMCs (same cells used in **C**) from non-responders patients pre-and post-RT by flow cytometry. **(F)** Representative flow cytometry analysis of YTHDF2 expression in MC38 tumor-infiltrating MDSCs (CD45^+^CD11b^+^Ly6C^hi^) three days after IR. Statistical analysis was performed using two-sided paired Student’s *t*-test (C, E). See also Figure S1.

Focusing on the myeloid compartment, we identified a monocytic MDSC cell subset (*Ly6c2*_Mono) in mice that dramatically increased in tumors post-IR (Figures 1A and S1E–S1F), characterized by low expression of *C1qa*, a macrophage marker, and high expression of *Ly6c2*, *CD11b* and *Arg1* (Figures 1B and S1F). This observation is consistent with previous work identifying Ly6C^+^ monocytes as MDSCs with superior T cell suppressive function.^26^ In an effort to translate this finding to humans, we evaluated the levels of MDSCs in PBMCs from cancer patients enrolled in a clinical trial at our institution; patients were treated with radiotherapy followed by pembrolizumab (anti–PD-1) (NCT02608385).^56^ MDSCs were significantly increased post-RT compared with matched pre-RT levels (*P*=0.029) in PBMCs. Notably, this increase was significant in patients who progressed at distal sites (outside of the radiation treatment fields; non-responders) (*P* = 0.04), but was not significantly changed in patients who did not progress at distal sites (responders) (*P* = 0.2) (Figure 1C). Consistent results were observed in PBMCs from metastatic NSCLC patients enrolled in another clinical trial (the COSINR study, NCT03223155^57^) at our institution (Figure S1G). In addition, we analyzed data sets in the TCGA database with MDSCs gene signature (*ARG1, CD14, CD44, CD40, S100A8, SELPLG, STAT6, TFRC, TGFB2, STAT3, CD274, ITGA3, SLA*, and *KDR*;^58^) to investigate whether MDSC associated genes are increased in clinical samples post-RT and whether the increase correlates to poor outcome. A low MDSC signature significantly associated with prolonged patient survival in a low grade glioma cohort with RT (*P*=0.01) and in a glioblastoma cohort with RT (*P*=0.049) (Figure S1H). These data indicate that radiation-induced MDSCs may be associated with worse clinical outcome in patients who receive local radiotherapy.

In addition to increased numbers of immunosuppressive MDSCs post-RT, we also investigated the impact of IR on epitranscriptomic modifications driven by RNA m^6^A methylation, which we have recently shown also modulates host antitumor immunity^50^. We observed that the expression of YTHDF2, a m^6^A reader protein, was dramatically elevated in MDSCs of irradiated versus untreated tumors (Figure 1D). In the PBMCs of patients with distal tumor progression, the YTHDF2 level in MDSCs was significantly increased post-RT compared with matched pre-RT samples/values (*P* =0.03, Figure 1E). Consistently, YTHDF2 protein level was markedly induced in MC38 tumor-infiltrating MDSCs after IR treatment (Figure 1F), but not in other infiltrating immune cells (Figure S1I). We next interrogated the temporal response of YTHDF2 following IR and observed that YTHDF2 was markedly elevated in a time-dependent manner (Figure S1J). IR also elicited a direct effect on YTHDF2 expression, as evidenced by upregulated YTHDF2 in bone-marrow derived CD11b^+^Ly6C^+^ cells treated with IR in a time- and dose-dependent manner (Figure S1K). These results demonstrate that IR induces YTHDF2 expression in MDSCs in both clinical and preclinical settings.

### *Ythdf2* deficiency in myeloid cells improves response to radiotherapy

**Figure 2.**
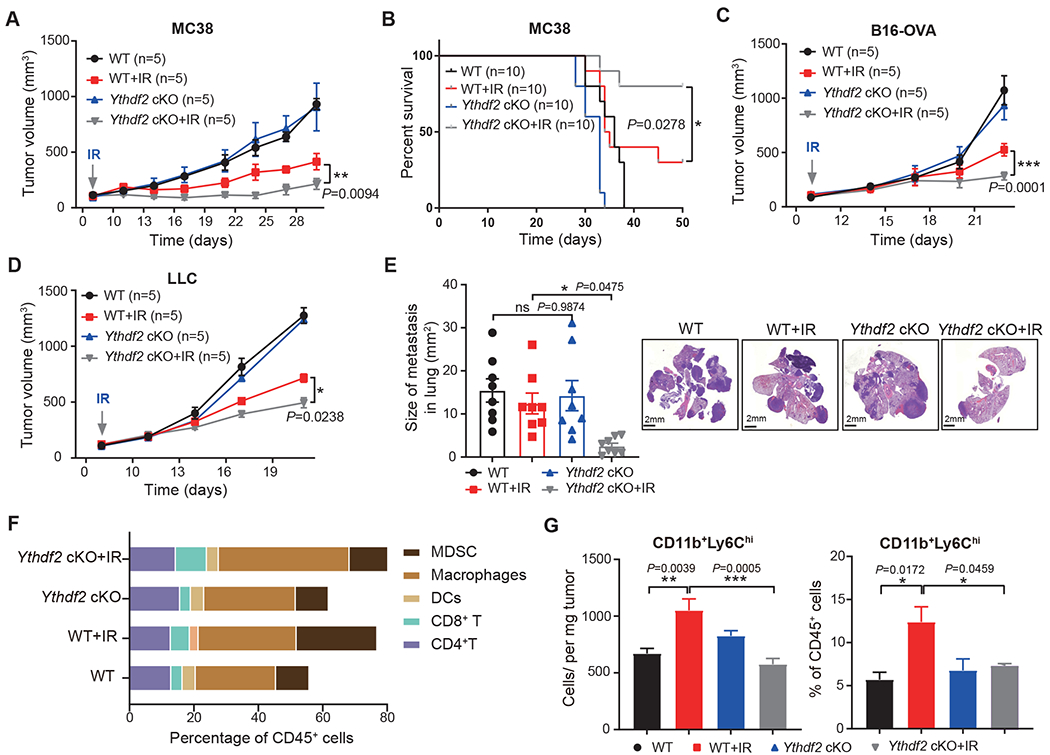
*Ythdf2* deficiency in myeloid cells improves response to radiotherapy. **(A, B)** Wild-type (*Ythdf2*^fl/fl^) or *Ythdf2*-cKO (*Lyz*^cre^*Ythdf2*^fl/fl^) mice were injected subcutaneously with 1x10^6^ MC38 cells. When the tumor size reached 100 mm^3^, tumor-bearing mice were treated with tumor-local IR (20 Gy, one dose). Tumor growth (**A**) and survival were monitored (**B**). Mice with tumor volumes less than 2, 000 mm^3^ were considered to be surviving. **(C, D)** Wild-type or *Ythdf2*-cKO mice were injected subcutaneously with 1x10^6^ B16-OVA cells (**C**) or 1x10^6^ LLC cells (**D**). When the tumor size reached 100 mm^3^, tumor-bearing mice were treated with local IR (20 Gy, one dose). Tumor growth was monitored. **(E)** Lung metastasis in WT or *Ythdf2*-cKO mice 22 days after IR. Treatments as indicated in (**D**). Size of lung metastases was measured. (n = 8 per group) **(F)** Populations of MC38 tumor-infiltrating immune cells assessed by flow cytometry (treatment conditions as indicated). MDSC: CD45^+^CD11b^+^Gr1^+^; Macrophages: CD45^+^CD11b^+^F4/80^+^; DCs: CD45^+^CD11c^+^MHCII^+^; CD8^+^ T: CD45^+^CD8a^+^; CD4^+^ T: CD45^+^CD4^+^; (n = 3 per group) **(G)** The number (left) and percentage (right) of tumor-infiltrating CD45^+^CD11b^+^Ly6C^hi^ cells three days after IR, as assessed by flow cytometry. (n = 3-5 per group) Data are represented as mean ± s.e.m., n, number of mice. One of two or three representative experiments was shown. Statistical analysis was performed using two-way ANOVA test with corrections for multiple variables (A, C, D), two-sided log-rank (Mantel-Cox) test (B) or one-way ANOVA with Bonferroni’s multiple comparison tests (E, G). *P < 0.05, **P < 0.01, and ***p < 0.001. See also Figure S2.

We reasoned that increased YTHDF2 in myeloid cells could alter the response to radiotherapy. To test this, we employed *Lyz*^Cre+^;*Ythdf2*^fl/fl^ conditional knockout mice (hereafter *Ythdf2*-cKO) and *Ythdf2*^fl/fl^ (hereafter WT) in the C57BL/6J genetic background for tumor growth experiments. In the syngeneic murine colon carcinoma (MC38) model, primary tumor growth in WT and *Ythdf2*-cKO mice was similar (Figure 2A). By contrast, local irradiation treatment resulted in a pronounced inhibition of tumor growth in *Ythdf2*-cKO mice compared with WT mice, assessed by both tumor volume and animal survival (Figures 2A, 2B). We also irradiated melanoma and Lewis lung carcinoma (LLC) tumors in both WT and *Ythdf2*-cKO mice, and observed a similar phenotype (Figures 2C, 2D). Intriguingly, in the LLC spontaneous lung metastasis model, a reduced metastatic burden was observed in lungs of *Ythdf2*-cKO mice that received IR compared with WT mice that received IR (Figure 2E). Taken together, these data indicate that deleting *Ythdf2* in myeloid cells enhanced the efficacy of radiotherapy through an increase in both local and distal metastasis control.

To assess whether distinct myeloid cell subsets are implicated in tumor progression and antitumor immunity following IR, we first characterized the effects of *Ythdf2* deletion in the TME after IR by profiling the tumor-infiltrating immune cells in MC38 tumors using flow cytometry. MDSCs and CD8^+^ T cells exhibited significant changes (Figure 2F). In WT mice, both the absolute number and percentage of CD11b^+^Ly6C^hi^ cells (monocytic MDSCs) increased in irradiated tumors compared with controls. In *Ythdf2*-cKO mice, the level of MDSCs did not increase in irradiated tumors and remained similar to the level in non-irradiated tumors from WT mice (Figure 2G). The level of MDSCs in irradiated tumors from *Ythdf2*-cKO mice was significantly decreased both in absolute number (*P* = 0.0005) and percentage (*P* =0 .0459; Figure 2G) compared with irradiated tumors in WT mice. The results demonstrate that *Ythdf2* deletion in myeloid cells led to a reduction of tumor-infiltrating MDSCs.

To examine whether specific deletion of myeloid *Ythdf2* results in improved immune function of T cells, we first measured the numbers of T cells and observed that both total CD8^+^ T cells and cytotoxic CD8^+^ T cells (IFNγ^+^CD8^+^) were significantly increased in irradiated tumors in *Ythdf2*-cKO mice compared with those of irradiated tumors from WT mice (Figures S2A, S2B). ELISPOT assays measuring the IFN-γ secreting capacity of CD8^+^ T cells sorted from the irradiated tumors in *Ythdf2*-cKO mice consistently showed a significant increase of IFN-γ production (Figures S2C). We also found increased levels of both IFN-γ and tumor necrosis factor (TNF)-α (Figure S2D), representing enhanced cytotoxic function. The antibody-mediated depletion of CD8^+^ T cells completely abrogated the antitumor efficacy of IR in *Ythdf2*-cKO mice (Figure S2E). Considering that conditional knockout of *Ythdf2* did not affect the development of T cells in naive mice (Figures S2F, S2G), our findings indicate that the CD8^+^ T cells are essential for enhanced IR-induced tumor control likely due to decreased MDSCs in *Ythdf2*-cKO mice.

### IR reshapes the composition of MDSC populations in blood and tumors

**Figure 3.**
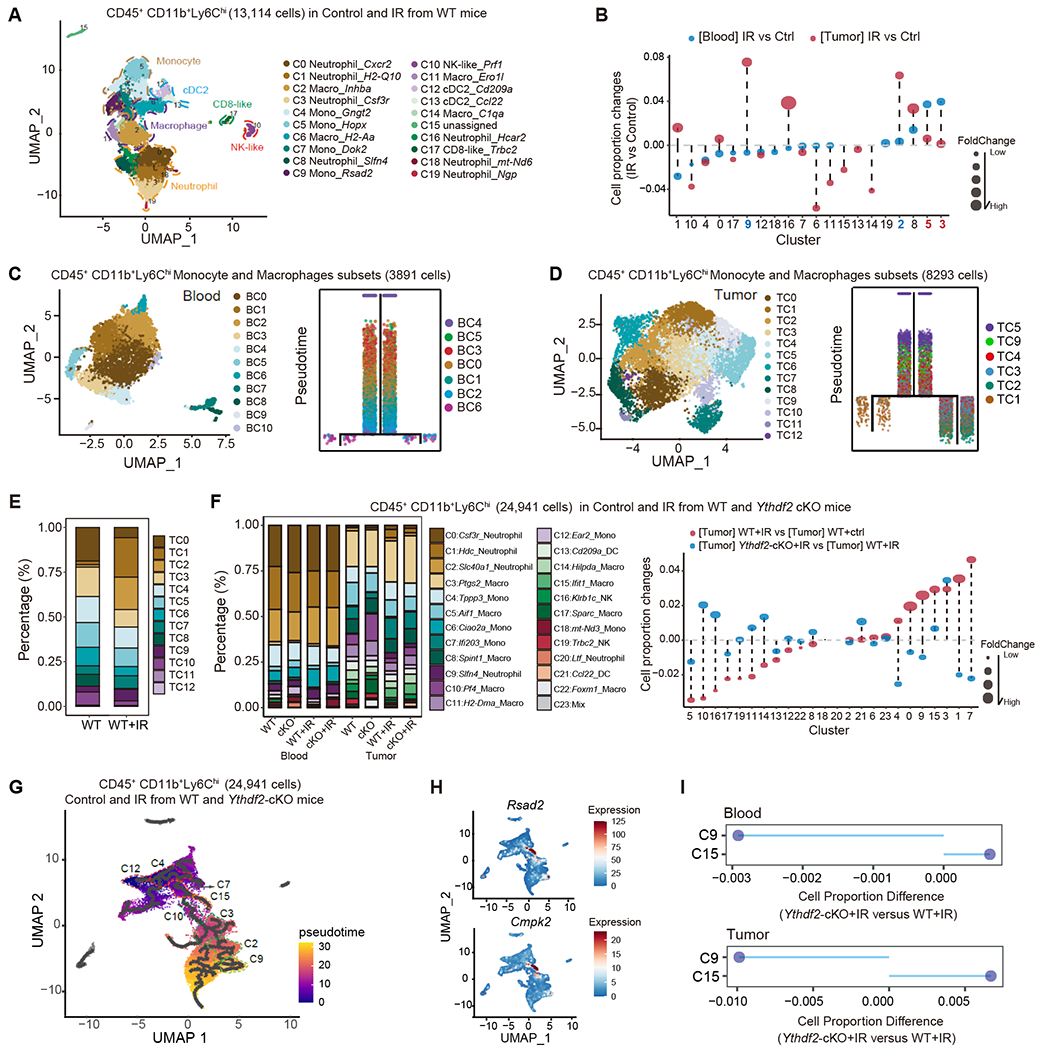
IR and YTHDF2 inhibition reshapes the composition of MDSC populations in blood and tumors. **(A)** UMAP plot displaying different mMDSCs-derived subsets from scRNA-seq. The CD45^+^CD11b^+^Ly6C^hi^ cells were sorted from blood and tumors in IR-treated MC38 tumor-bearing mice, respectively three days after IR. (Five mice were pooled per group). **(B)** Cell proportion changes (IR vs. non-IR) of different mMDSCs-derived subsets in blood and tumors, respectively. **(C)** Cell trajectory of cell populations in blood (only including monocytes and macrophages subsets) were visualized using UMAP. **(D)** Cell trajectory of cell populations in tumors (only including monocytes and macrophages subsets) were visualized using UMAP. **(E)** Proportion of different mMDSC-derived subsets in tumors with non-IR versus IR treatment. **(F)** Proportion of mMDSC-derived subsets in blood and tumors from WT and *Ythdf2*-cKO mice with non-IR versus IR treatment (left); Cell proportion changes of mMDSC-derived subsets in tumors in WT+IR *vs*. WT and *Ythdf2*-cKO+IR *vs*. WT+IR (right). **(G)** Cell trajectory of combined cell populations in blood and tumors from WT or *Ythdf2*-cKO mice. **(H)** Expression level of gene signatures of C15 in UMAP space. **(I)** Proportion of C15 and C9 clusters from blood and tumors, respectively (*(Ythdf2*-cKO+IR versus WT+IR). See also Figures S3, S4, and Tables S1, S2.

We aimed to further delineate the effects of YTHDF2 on MDSCs in the context of IR. We first investigated the state of monocytic MDSCs (mMDSC), which are immature myeloid cells, by performing scRNA-seq using CD45^+^CD11b^+^Ly6C^hi^ cells (mMDSC) isolated from blood and MC38 tumors of irradiated mice. To dissect mMDSC heterogeneity, we applied unbiased clustering algorithms and identified 19 distinct cell populations belonging to four broad cell types: monocytes, macrophages, DCs, and neutrophils (Figures 3A and S3A). In blood, IR consistently modulates mMDSC development. For example, C3 (Neutrophil-*Cf3r*) and C5 (Mono-*Hopx*) (labeled blue) were increased in blood after IR; C9 (Mono-*Rsad2*) and C2 (Macro-*Inhba*) (labeled red) were increased in tumors after IR (Figure 3B). Local tumor IR modulates mMDSC development intratumorally and systemically, demonstrating that local IR alters systemic immune responses.

To infer the potential differentiation trajectories, we compiled monocyte and macrophage subsets of mMDSCs derived from blood along a pseudotime axis and observed that the differentiation occurs on a tightly organized trajectory, starting from BC4 cluster, through BC0, BC1, BC2, and ending with the BC6 cluster (Figure 3C). BC6 exhibits cluster-specific expression of *Ccnb2, Birc5, Stmnl, Pclaf, Cdca3, Mki67*, and *Cks1b* (Figure S3B), indicative of highly proliferative activity. In tumors, we also zoomed in monocyte and macrophage subsets and found that cluster TC5 gradually develops into TC9, then TC1; or into TC2, and TC3 (Figure 3D). Notably, the immunosuppressive gene *Arg1* was highly expressed in ten out of thirteen clusters, indicative of a suppressive phenotype (Figure S3C, Table S1). The proportion of TC1 and TC2 cells, annotated by gene expression signature as inflammatory and suppressive mMDSC population, respectively, were increased following IR compared to non-IR condition (Figure 3E). IR also resulted in decrease of several populations in tumor mMDSCs, such as TC0, annotated with high ribosomal activity, and TC3 which exhibits the expression of MHC class-associated genes (*H2-Ab1, H2-Aa, H2-Aa*, and *H2-Eb1*), suggesting that it could be classified as TAM with cross-presentation activity. To investigate the role of IR in the differentiation of mMDSC in blood, we next quantified the pseudotime of each cell subset in irradiated and unirradiated mice. We found that BC0 and BC1 from irradiated mice showed increased pseudotime compared with unirradiated controls (Figure S3D). We also observed significantly increased pseudotime of TC9, TC2 and TC3 from irradiated mice versus unirradiated controls (Figure S3E). Together, our findings reveal that tumor-local IR remodeled the landscape of mMDSC populations, possibly through accelerating mMDSC differentiation, and triggered a suppressive tumor microenvironment.

### YTHDF2 affects mMDSC differentiation in the context of IR

To assess the relationship between YTHDF2 inhibition and mMDSC differentiation and to obtain a full picture of the population change, we queried scRNA-seq data of mMDSCs (all CD45^+^CD11b^+^Ly6C^hi^ cells) from *Ythdf2*-cKO mice. *Ythdf2* knockout led to changes in the proportion of distinct mMDSC-derived subsets in both blood and tumor in irradiated and unirradiated controls, compared with WT mice (Figures 3F and S3F–S3G). First, we conducted the trajectory analysis of mMDSC-derived subsets in blood and observed that, in unirradiated mice, BC4 and BC1 showed increased pseudotime in *Ythdf2*-cKO mice compared with WT (Figure S3H). In irradiated mice, BC2 showed increased pseudotime in *Ythdf2*-cKO mice compared with WT (Figure S3I). Second, we conducted the trajectory analysis of mMDSC-derived subsets in tumor and found that TC5 and TC3 showed a significantly decreased pseudotime in *Ythdf2*-cKO compared with the WT (Figure S3J). TC5 and TC2 showed decreased pseudotime in *Ythdf2*-cKO+IR compared with the WT+IR (Figure S3K). Strikingly, the pattern of cell population changes in “*Ythdf2*-cKO+IR *vs*. WT+IR” is largely opposite to that in “WT+IR *vs*. WT” (Figure 3F), suggesting YTHDF2 plays a key role in MDSC differentiation in response to IR. By establishing a single-cell atlas, we demonstrated that *Ythdf2* knockout alters the mMDSC differentiation and the effects are amplified by IR.

MDSCs migrate from blood to tumor in response to radiotherapy. To depict a continuous picture of mMDSC differentiation, we conducted the trajectory analysis in mMDSCs combining all samples (blood and tumor from WT or *Ythdf2*-cKO +/− IR treatment) (Figures 3G and S3F). We identified C12 as monocyte precursor on the basis of abundant expression of *Ear2*.^59^ The C12 population evolves into C4, and then C7, followed by branching into two separate paths: 1) C15 and 2) from C10, C3, C2, to C9 (Figure 3G). Among them, C12 and C4 mainly reside in blood, and C7 mainly associates with tumor (Figure S3L, Table S2). C15 is classified as an M1-like macrophage, characterized by high levels of *Rsad2* and *Cmpk2* (Figure 3H). C3, C2, and C9 were characterized as polymorphonuclear(PMN)-MDSCs-like cells, consistent with a concept that mMDSCs can differentiate into PMN-MDSCs.^60^ The percentage of C15 (M1-like cells) significantly increased, while C9 (PMN-MDSC-like cells) significantly decreased in IR treated *Ythdf2*-cKO mice *versus* IR treated WT mice (Figure 3I), as further confirmed by flow cytometry analysis (Figure S4A); this data mirrors the tumor growth phenotypes. These modifications of mMDSC-derived clusters resulted in reprogramming of the host immune microenvironment locally and systemically in favor of enhanced anti-tumor immunity in *Ythdf2*-cKO mice in response to IR.

### YTHDF2 controls MDSC migration and suppressive function in the context of IR

**Figure 4.**
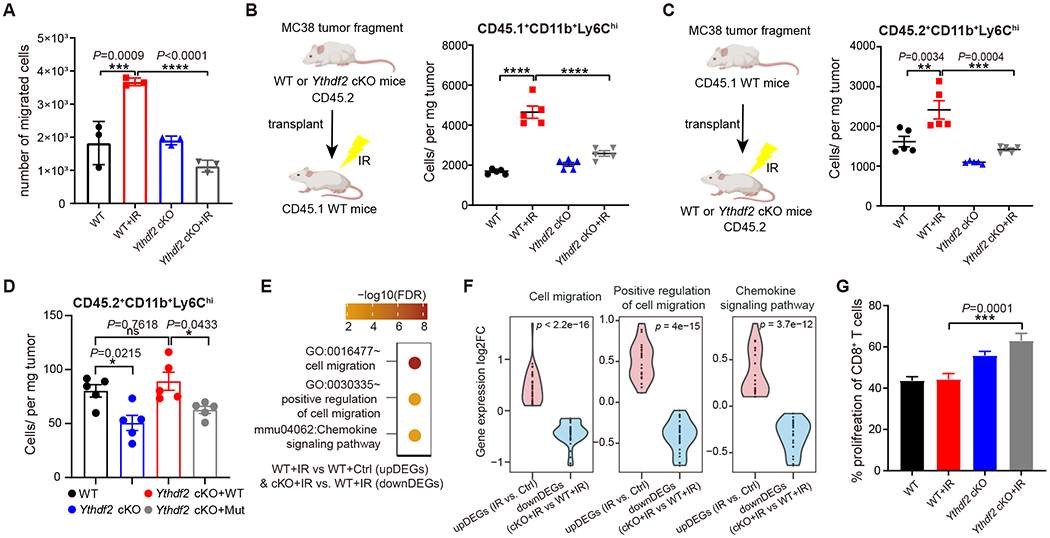
YTHDF2 controls MDSC migration and suppressive function in the context of IR. **(A)** MDSCs were sorted from MC38 tumors, as indicated in (Fig. 2A), and subjected to the trans-well migration assay. Migrated cells on the trans-well membranes were visualized under a light microscope and quantified. (n = 3 per group) **(B)** MC38 tumor fragments from WT or *Ythdf2*-cKO mice (CD45.2) were transplanted into CD45.1 WT mice. Three days later, tumors were treated with local tumor IR (20 Gy, one dose). Three days after IR, the number of tumor-infiltrating CD45.1^+^CD11b^+^Ly6C^hi^ cells was determined by flow cytometry. (n = 5 per group) **(C)** MC38 tumor fragments from CD45.1 WT mice were transplanted into WT or *Ythdf2*-cKO mice (CD45.2). Three days later, tumors were treated with local tumor IR (20 Gy, one dose). Three days after IR, the number of tumor-infiltrating CD45.2^+^CD11b^+^Ly6C^hi^ cells was determined by flow cytometry. (n = 5 per group) **(D)** The YTHDF2 (*Ythdf2*-WT) and m^**6**^ A-binding-site-mutated YTHDF2 (*Ythdf2*-Mut) overexpressing *Ythdf2*-cKO BM-MDSCs (CD45.2) were adoptive transfer into MC38 tumor-bearing CD45.1 mice. On the same day, mice were treated with local tumor IR (20 Gy). Three days after IR, the number of tumor-infiltrating CD45.2^+^CD11b^+^Ly6C^hi^ cells was determined by flow cytometry. (n = 5 per group) **(E)** CD11b^+^ myeloid cells were sorted from MC38 tumors, as indicated in Fig. 2A, and subjected to bulk mRNA-seq. Heatmap of functional enrichment analysis of differentially expressed gene pathways. **(F)** Violin plot of gene expression fold changes (log2FC) in genes related to chemokine signaling pathways, cell migration, and positive regulation of cell migration pathways (comparing WT+IR versus WT+Control, and *Ythdf2*-cKO + IR versus WT + IR). **(G)** Flow cytometry analysis of an *in vitro* proliferation assay showing the frequency of proliferating CD8^+^ T cells when co-cultured with MDSCs sorted from different MC38 tumors, as indicated. (n = 3 per group) Data are represented as mean ± s.e.m., n, number of mice. One of two representative experiments was shown (A-C). Statistical analysis was performed using one-way ANOVA with Bonferroni’s multiple comparison tests (A-D, G). *P < 0.05, **P < 0.01, ***P < 0.001, and ****P < 0.0001. See also Figure S4.

Having demonstrated that YTHDF2 affects MDSC differentiation in the context of IR, we next investigated the role of YTHDF2 in IR-induced MDSC migration. *Ythdf2* deletion impaired the migratory capacity of MDSCs as evidenced by a migration assay in which MDSCs in irradiated tumors of *Ythdf2*-cKO mice showed a significantly lower migration compared with that of WT mice (Figure 4A). To further verify this, MC38 tumor fragments from WT or *Ythdf2*-cKO mice (both of which are CD45.2), which contain pre-existing MDSCs, were harvested and inoculated into CD45.1 WT mice. The CD45.1 mice were treated with IR. Three days after IR, there was no significant increase of tumor-infiltrating CD45.1^+^CD11b^+^Ly6C^hi^ (mMDSC) cells post-IR in tumors derived from *Ythdf2*-cKO mice, whereas a significant increase in this population was observed in tumors derived from WT mice post-IR (Figure 4B). Further, we observed consistent changes in chemokine expression in the infiltrating MDSCs (Figure S4B). These results suggest that loss of *Ythdf2* in myeloid cells abrogated IR-induced enhanced chemokine production to attract further infiltration of MDSCs. We also inoculated MC38 tumor fragments grown in CD45.1-WT mice into WT or *Ythdf2*-cKO mice (CD45.2) and found that IR failed to induce the accumulation of tumor-infiltrating CD45.2^+^ MDSCs in *Ythdf2*-cKO mice (Figure 4C), suggesting that *Ythdf2*-cKO MDSCs were less effective in trafficking to the tumor following IR, possibly due to the decrease of certain chemokine receptor expression (Figure S4B). To further demonstrate that the effect of YTHDF2 on MDSC migration is dependent on its m^6^A binding, we force expressed YTHDF2 (*Ythdf2*-WT) and m^6^A-binding-site-mutated YTHDF2 (*Ythdf2*-Mut) in *Ythdf2*-deficient BM-MDSCs (CD45.2) and adoptively transferred these cells into MC38 tumor-bearing CD45.1 mice followed by IR treatment. Three days post-IR, the number of newly-infiltrated CD45.2 MDSCs in tumors were analyzed. Compared to transferred WT-MDSCs, *Ythdf2*-cKO-MDSCs elicited significantly lower migration capacities post-IR (**P**=0.0215). YTHDF2-WT overexpressing *Ythdf2*-cKO-MDSCs rescued the migration (*Ythdf2*-cKO+WT *vs*. WT, *P*=0.7618), whereas YTHDF2-Mut overexpression could not rescue migration (*Ythdf2*-cKO+WT *vs*. *Ythdf2*-cKO+Mut, *P*=0.0433) (Figure 4D). Taken together, our results indicate that deletion of *Ythdf2* in the myeloid compartment leads to defects in both migration capacity and chemoattraction of MDSCs following IR, and the phenotype requires the m^6^A binding capacity of YTHDF2.

For validation of these findings, we performed mRNA-seq using MC38 tumor-infiltrating CD11b^+^ myeloid cells in WT and *Ythdf2*-cKO mice +/− IR. As expected, the GO enrichment analysis indicated that three pathways, including those affecting cell migration, chemokine signaling, and positive regulation of cell migration, were up-regulated in WT+IR versus WT+ctrl, and were down-regulated in *Ythdf2*-cKO+IR versus WT+IR (Figures 4E–4F). These findings support our observation of MDSC migration phenotypes *in vivo*.

In terms of suppressive function, we observed that, in the context of IR, tumor-infiltrating *Ythdf2*-cKO MDSCs exhibited attenuated suppressive function during co-culture with activated naïve CD8^+^ T cells, compared with WT MDSCs (Figure 4G). In pursuing this further, we investigated the expression levels of proteins produced by MDSCs that mediate immune suppression. IR significantly induced IL-10 production and *Arg1* expression in tumors in WT mice compared with *Ythdf2*-cKO mice (Figures S4B–S4C). Taken together, these results reveal that *Ythdf2* knockout impairs both MDSC migration and suppressive functions, which may be critical to the enhanced antitumor effect of IR observed in *Ythddf*-cKO.

### NF-κB/RELA mediates radiation-induced YTHDF2 expression in MDSCs

We sought to investigate the potential mechanisms involved in radiation induction of YTHDF2 in MDSCs. First, we performed functional enrichment analysis with genes differentially expressed in “monocytic MDSC_*Lydc2*” (P01 population, Figure 1A) following IR treatment, and found that genes of the NF-kappa B (NF-κB) signaling pathway are enriched in this population post-IR (Figure S4D). These data are consistent with previous findings that IR activates NF-κB.^61–63^ Further investigation of the relationship between NF-κB and YTHDF2 revealed that the level of nuclear RELA is increased after IR (Figure S4E). YTHDF2 expression was not induced by IR in MDSCs deficient of *Nfkb1* (Figure S4E), demonstrating that NFKB1 is required for IR induction of YTHDF2. NFKB1 is an important component in NF-κB signaling by forming a RELA/NFKB1 heterodimer required for RELA nuclear translocation.^64^ We next analyzed a public dataset of RELA chromatin immunoprecipitation sequencing (ChIP-seq) conducted in mouse bone marrow-derived macrophages and found the predicted direct binding between RELA and the *Ythdf2* promoter region (Figure S4F). To verify this finding, we performed ChIP coupled with quantitative PCR (ChIP-qPCR) analysis using bone marrow-derived CD11b^+^Ly6C^+^ cells. The results revealed that RELA indeed directly binds to the *Ythdf2* promoter region (~1.0-2.0 kb proximal to the transcription start site) (Figure S4G). Collectively, these findings indicate that IR upregulates YTHDF2 expression via the NF-κB/RELA signaling pathway.

### IR-induced YTHDF2 enhances NF-kB signaling by promoting m^6^A-modified RNA degradation

**Figure 5.**
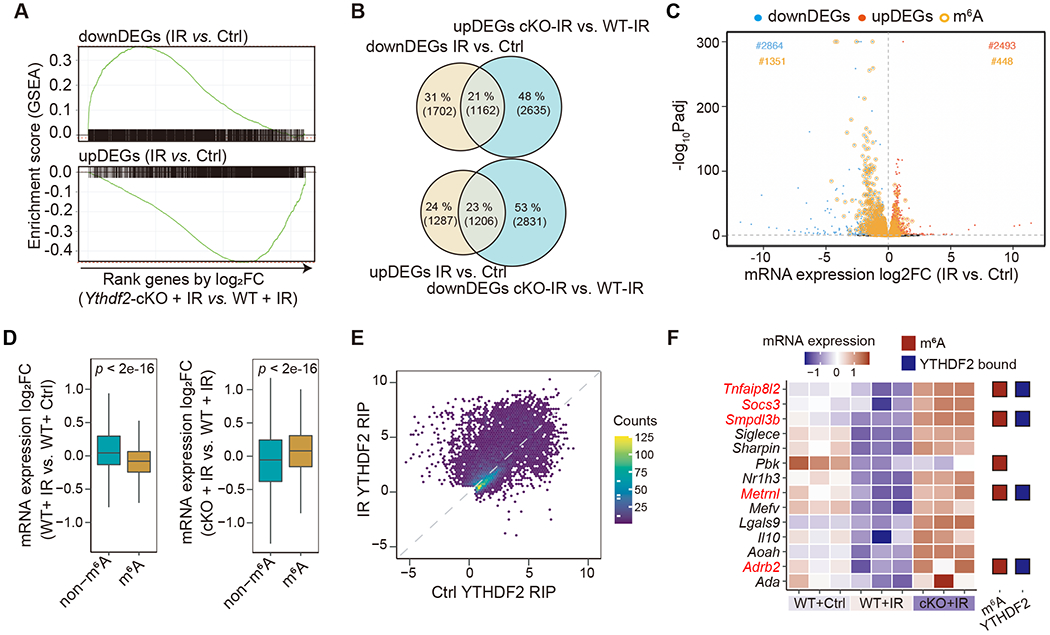
IR-induced YTHDF2 enhances NF-kB signaling by promoting m^6^A-modified RNA degradation. **(A)** The tumor-infiltrating CD11b^+^ myeloid cells were isolated from MC38 tumor-bearing WT or *Ythdf2*-cKO mice with IR or unirradiated controls three days after IR followed by bulk RNA-seq. Gene Set Enrichment Analysis (GSEA) of differentially expressed genes following IR treatment (IR *vs*. Ctrl) against ranked list of genes according to expression changes comparing *Ythdf2*-cKO+IR versus WT+IR. **(B)** Venn diagram of overlapping genes that were downregulated following IR vs. Ctrl and upregulated following *Ythdf2*-cKO+IR vs. WT+IR (top); or upregulated upon IR vs. ctrl and downregulated upon *Ythdf2*-cKO +IR vs. WT+IR (bottom). **(C)** Volcano plot of genes with differential expression levels in the tumor-infiltrating CD11b^+^ myeloid cells (IR vs. Ctrl). m^6^A marked genes are shown with orange circles. Downregulated genes (downDEGs) are highlighted with blue and upregulated genes (upDEGs) with red. CD11b^+^ myeloid cells were collected from five pooled MC38 tumor-bearing mice three days after IR. **(D)** Boxplot showing gene expression log2FC comparing WT+IR vs. WT+ctrl (left); and *Ythdf2*-cKO+IR vs. WT+IR (right). Genes were categorized into two groups according to whether they were marked with m^6^A or not (non-m^6^A). For box plots, the center lines represent the medians, the box show the upper(top) and lower(bottom) quartiles, vertical lines represent 1.5x the interquartile ranges. *P* values were calculated by the nonparametric Wilcoxon-Mann-Whitney test. **(E)** The tumor-infiltrating CD11b^+^ myeloid cells were collected from five pooled MC38 tumor-bearing mice three days after IR followed by RIP-seq. Scatter plot of YTHDF2 binding intensity on its target genes (Ctrl *vs*. IR). **(F)** Heatmap showing gene expression level in WT mice with non-IR (WT+Ctrl) and IR (WT+IR) treatment, and *Ythdf2*-cKO mice with IR treatment (cKO+IR) (left). Genes were further categorized into groups according to whether they were bound by YTHDF2, or marked with m^6^A (right). See also Figure S5.

To investigate the molecular mechanisms of YTHDF2 function in MDSCs in the context of IR, we reanalyzed the mRNA-seq of MC38 tumor-infiltrating CD11b^+^ myeloid cells in *Ythdf2*-cKO mice with IR or unirradiated controls. We analyzed the gene expression profiles and found that knockout of *Ythdf2* abolished the transcriptional changes induced by IR alone (Figure 5A). We focused on the differentially expressed genes, comparing IR versus non-IR in WT mice and IR versus non-IR in *Ythdf2*-cKO mice (Figure 5B) to perform gene enrichment analysis. We found an enrichment of the “negative regulation of inflammatory response” pathway in *Ythdf2*-cKO+IR (Figure S5A), containing five genes (*Tnfaip8l2*, *Socs3, Smpdl3b*, *Metrnl* and *Adrb2*) which have been reported as negative regulators for NF-κB signaling,^65–69^ which facilitates MDSC migration and chemokine/cytokine regulation.^70^ Thus our data indicate that IR induces YTHDF2 via NF-κB, and the elevated YTHDF2 levels may in turn enhance NF-κB signaling in MDSCs, thus forming an IR-YTHDF2-NF-κB circuit.

To test this hypothesis, we sought to characterize the downstream direct targets of YTHDF2 by performing *N*^6^-methyladenosine-sequencing RNA immunoprecipitation followed by high-throughput sequencing (MeRIP-seq) and RNA immunoprecipitation sequencing (RIP-seq). We observed that a majority of m^6^A-marked genes were down-regulated after IR treatment (1351 down-regulated genes versus 448 up-regulated genes) (Figure 5C) and *Ythdf2* deletion reversed this suppression (Figures 5D and S5B). Furthermore, with YTHDF2 expression being elevated by IR, the number of YTHDF2-bound transcripts increased accordingly (Figures 5E and S5C–S5D), suggesting that increased YTHDF2 binding post IR mediated the decrease of mRNA abundance.

Close investigation of the above-mentioned genes as negative regulators of NF-κB signaling revealed three genes (*Adrb2, Metrnl*, and *Smpdl3b*) are m^6^A marked and targeted by YTHDF2 (Figures 5F and S5E–S5F). Additionally, their transcript half times were increased in *Ythdf2*-cKO (Figure S5G), consistent with the known function of YTHDF2 in promoting mRNA degradation. We hypothesized that, in the context of IR, *Ythdf2* enhances NF-κB signaling through reducing the expression of *Adrb2, Metrnl*, or *Smpdl3b*. To test this, we generated BM-MDSCs with all 3 genes knocked down (3xKD) (Figure S5H) and conducted western blot assay for NF-κB signaling. We observed increased levels of IκBα phosphorylation and nuclear localization of RELA in knockdown MDSCs (Figure S5I). Moreover, IκBα phosphorylation inhibitor (BAY 11-7082)^71^ treatment prevented this increase(Figure S5I). These results indicate that the m^6^A/YTHDF2 axis regulates NF-κB signaling in MDSCs by targeting negative regulators of the NF-κB pathway.

To assess whether the IR-YTHDF2-NF-κB axis contributes to MDSC migration or function, we employed *Ccr2*-KO mice in which the radiation-induced MDSC infiltration into tumors is markedly decreased^26^. We adoptively transferred 3xKD-MDSCs into MC38 tumor-bearing *Ccr2*-KO mice and observed that compared with the transferred WT-MDSCs, 3xKD-MDSCs elicited significantly higher migration capacities post-IR (Figure S5J). *In vitro* migration assays of 3xKD-MDSCs also showed similar results (Figure S5K). To further confirm the involvement of YTHDF2 in radiation-induced MDSC infiltration, we performed the aforementioned transfer experiment using 3xKD-*Ythdf2*-cKO-MDSCs. As expected, three days after IR, the level of infiltrating MDSCs (CCR2^+^CD11b^+^Ly6C^hi^) was restored to a level similar to 3xKD-MDSCs transfer (Figure S5L). Functionally, we observed consistent changes in the expression of genes associated with migration and function of MDSCs, including *Ccl2*, *Ccl5, Cxcl16, Ccr7*, and *Il10* (Figure S5M). Collectively, these data demonstrate that the enhanced migration and function of MDSCs induced by IR rely on the expression of YTHDF2, most likely via enhanced activation of the NF-κB pathway. Together with the previous finding that IR-mediated YTHDF2 induction relies upon NF-κB activation, we propose that an YTHDF2-NF-κB positive feedback loop governs migration and suppression of MDSCs and tumor extrinsic radioresistance.

### Pharmacological inhibition of YTHDF2 enhances responses to radiotherapy and immunotherapy

To demonstrate that enhanced efficacy of RT by YTHDF2 deficiency can be translated into a clinically relevant strategy, we screened an in-house compound library with fluorescence polarization based high-throughput screening assays and found a small molecule, DC-Y13, as an inhibitor of YTHDF2. DC-Y13 inhibits YTHDF2 binding to m^6^A-containing RNA with an IC_50_ of 74.6 ± 1.9 μM measured via AlphaScreen assay (Figure S6A). To improve its inhibitory activity, we designed and synthesized DC-Y13-27, a derivative of DC-Y13, of which IC_50_ was determined to be 21.8 ± 1.8 μM (Figure S6A). To further assess the capacity of DC-Y13-27 binding to YTHDF2, we conducted a microscale thermophoresis assay and confirmed that DC-Y13-27 binds to YTHDF2 with a binding constant (KD) of 37.9 ± 4.3 μM (Figure S6B). We further confirmed the specific binding using surface plasmon resonance (SPR) assay (Figure S6C). Using the mRNA level of a direct binding target of YTHDF2 (*PRR5*)^72^ as readout, we further validated that DC-Y13-27 increased transcript level of YTHDF2 target at a similar level as that in *YTHDF2* knockdown (Figure S6D). We next explored the inhibitory activity of DC-Y13-27 against YTHDF1, another member of the YT521-B homology (YTH) domain-containing proteins family to evaluate its selectivity. DC-Y13-27 obstructs the interaction between YTHDF1 and m^6^A with an IC_50_ of 165.2 ± 7.7 μM, as shown in Figure S6E. Biologically, the inhibitor did not inhibit the expression of LRPAP1, a reported YTHDF1 target^73^ (Figure S6F), suggesting that DC-Y13-27 exhibits a preference for inhibiting YTHDF2 binding to m^6^A-modified RNA.

**Figure 6.**
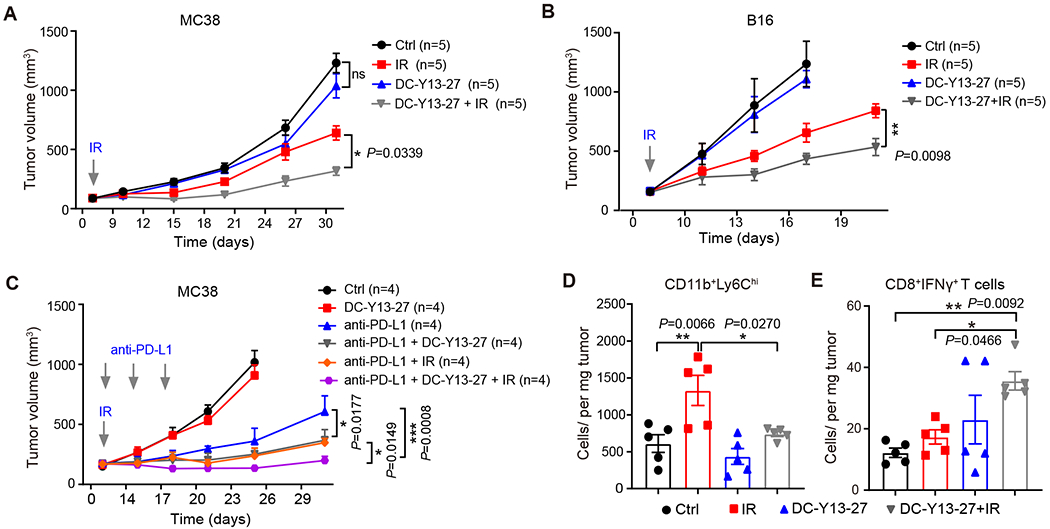
Pharmacological inhibition of YTHDF2 enhances responses to radiotherapy and immunotherapy. **(A, B)** WT mice were injected subcutaneously with 1x10^6^ MC38 cells (**A**) or 1x10^6^B16F1 cells (**B**). When the tumor size reached 100 mm^3^, tumors were treated with local IR (20 Gy, one dose). On the same day, the mice were treated with DC-Y13-27 (9 μg/per mice, daily) until the end of the experiment. Tumor growth was monitored. **(C)** WT mice were injected subcutaneously with 1x10^6^ MC38 cells. When the tumor size reached 100 mm^3^, tumors were treated with local IR (20 Gy, one dose), anti-PD-L1 antibody (2 doses per week, three doses total) and/or DC-Y13-27 (9 μg/per mice, daily), as indicated. Tumor growth was monitored. **(D)** The numbers of tumor-infiltrating CD45^+^CD11b^+^Ly6C^hi^ cells in mice, with different treatment as indicated, were assessed by flow cytometry three days after IR. (n = 5 per group) **(E)** The numbers of tumor-infiltrating CD45^+^CD8^+^IFNγ T cells in MC38 tumor-bearing mice with treatments as indicated seven days after IR. (n = 5 per group) Data are represented as mean ± s.e.m., n, number of mice. One of two representative experiments was shown. Statistical analysis was performed using two-way ANOVA test with corrections for multiple variables (A-C) or one-way ANOVA with Bonferroni’s multiple comparison tests (D, E). *P < 0.05 and **P < 0.01. See also Figure S6.

To investigate whether inhibition of YTHDF2 improves the response to IR at a similar level as *Ythdf2* genetic deletion, we first confirmed that the inhibitor was able to inhibit NF-κB activation (Figure S6G). Tumor-bearing mice were treated daily with the proof-of-principle compound DC-Y13-27 starting on the day of IR treatment. Consistent with results obtained in *Ythdf2*-cKO, DC-Y13-27 treatment alone did not inhibit tumor growth in either the MC38 or B16 murine models. However, when combined with IR treatment, DC-Y13-27 treatment significantly enhanced tumor growth inhibition of IR compared with IR alone (Figures 6A–6B). We also investigated whether DC-Y13-27 can further increase efficacy of IR and anti-PD-L1 treatment using the MC38 model. Compared with any single treatment group, combination treatment with DC-Y13-27 and anti-PD-L1 resulted in significantly slower growth of MC38 tumors, and the triple therapy of DC-Y13-27, IR, and anti-PD-L1 gave rise to the most robust antitumor effects (Figure 6C).

To interrogate the underlying immunological mechanisms, we profiled the tumor-infiltrating immune cells in MC38 tumors following DC-Y13-27 treatment by flow cytometry. We observed that IR failed to increase infiltration of CD11b^+^Ly6C^hi^ cell in tumors with DC-Y13-27 treatment, whereas a 2-fold increase was observed following IR alone (Figure 6D). This is consistent with our aforementioned findings in irradiated *Ythdf2*-cKO mice. Given the critical roles of MDSCs in determining T-cell infiltration and function, we examined the CD8^+^ T-cell populations in tumors five days after IR and DC-Y13-27 treatment. The total numbers of both CD8^+^ T cells and IFNγ CD8^+^ T cells were increased in tumors receiving the combination treatment (Figures 6E and S6H). This was also evidenced by the abolished antitumor efficacy of DC-Y13-27 plus IR in *Rag1* knockout mice (Figure S6I). The results demonstrate that the antitumor effect of the combination treatment with YTHDF2 inhibition and IR relies on adaptive immunity and is similar to that observed in *Ythdf2* deleted mice.

## Discussion

Here we report that the genetic and proof-of-principle pharmacologic blockade of the m^6^A reader YTHDF2 improves local radiotherapy and combined radio-immunotherapy effects by reshaping the MDSC compartment, and inhibiting MDSC migration and suppressive functions. YTHDF2 was rapidly induced via IR-activated NF-κB/RELA, suggesting that YTHDF2 may play a critical role in the response to radiation-induced stress. Our study delineates a previously unknown link between IR stress and RNA m^6^A modification. YTHDF2 triggers degradation of transcripts encoding the negative regulators of IκBα, leading to enhanced NF-κB signaling, resulting in a positive feedback loop to sustain YTHDF2 expression. The IR-YTHDF2-NF-κB circuit in MDSCs represents a previously unrecognized mechanism of extrinsic radioresistance.

Through the use of single cell RNA-sequencing, we are able to describe the murine colon tumor (MC38)-infiltrating immune cell atlas in the context of IR and also provide a reference map of differentiating and mature myeloid transcriptional states in the context of ablative IR (20 Gy). Based on the trajectory and functional gene enrichment analysis, myeloid cells differentiate/mature into distinct subpopulations as a response to radiation stress and these likely exert different functions compared with myeloid cells in a steady state. Following IR, *Ythdf2* knockout affects the differentiation in both tumors and blood and thereby confers a unique mMDSC landscape. Considered together, our data suggest that the IR-YTHDF2 axis plays a critical role in regulating mMDSC differentiation. We acknowledge that without in-depth lineage studies, we cannot confirm the changes in development/differentiation trajectory of mMDSC in blood and tumor in relation to YTHDF2 status and radiation. Elucidation of the exact function of myeloid subpopulations and the molecular bases of heterogeneity varied in different cancer types responding to different treatments would require future investigations.

Our finding that NF-κB signaling in MDSCs is controlled in a positive feedback loop by YTHDF2, provides a fresh link between RNA m^6^A modification (YTHDF2 reader protein) and NF-κB signaling in specific immune cell populations. The RNA-seq and m^6^A target analyses provide the clues regarding the roles of YTHDF2 and NF-κB signaling in the regulation of MDSC migration and suppressive functions. The three YTHDF2 direct targets identified in this study serve as negative regulators of NF-κB signaling and may play additional roles in related biological processes.^65,74,75^ We cannot rule out the possibility that these three proteins may target additional signaling involved in the regulation of MDSC suppressive functions. We showed here that NF-κB plays a central role in the YTHDF2-regulated MDSC function, however, other signaling pathways, such as TNF signaling, may also contribute to our observed phenotypes. We demonstrated that IL-10 was upregulated by IR in an YTHDF2-NF-κB dependent manner in MDSCs. The induced IL-10 might in turn affect NF-κB signaling since it has been shown to inhibit NF-κB activity in monocytes.^76^ We hypothesize that IL-10 may act in a negative feedback loop to regulate NF-κB in an YTHDF2 dependent manner, as a part of intricate network of biological responses to inflammation. The function and roles of YTHDF2 need to be further explored in other types of immune cells and/or in the context of distinct conditions.

Considering that YTHDF2 is expressed in most immune cells at different levels, we speculate that YTHDF2 may affect functions of these different immune cells and YTHDF2 depletion in different immune cell types may impact host tumor immune response differently. For this reason, we cannot rule out the possibility that the improved antitumor effects of YTHDF2 inhibitor with radiotherapy or immunotherapy might be due to its action in other types of immune cells. Our results provide proof-of-principle preclinical evidence that YTHDF2 inhibition with a selective small molecule inhibitor in a whole-animal setting notably improves the antitumor efficacy of radiotherapy, anti-PD-L1 immunotherapy or the combination. Therefore, the pharmacological inhibition of YTHDF2 *in vivo* could potentially complement and synergize with many existing cancer therapies to overcome immunosuppression and enhance treatment efficacy and patient response rates. We concede that DC-Y13-27 is a tool compound that only showing high selectivity, which is similar to one published m^6^A-assosicated inhibitor (METTL13 inhibitor, STM2457)^77^. We wish to develop more potent inhibitors for clinical translation in the future.

Genetic YTHDF2 depletion not only enhances the local anti-tumor effects of radiation but also suppresses distant metastasis that may occur through radiation induced MDSC mobilization. The clinical importance of these observations is significant; ablation of YTHDF2 activity could be an ideal strategy of enhancing the effects of local radiotherapy as well as suppression of distant metastasis. Radiation-induced YTHDF2 expression may also explain the failure to induce a consistent abscopal effect—a rare phenomenon involving antitumor effect on distant metastasis following irradiation of a single lesion—as well as the failure to improve survival in many radiotherapy/checkpoint inhibitor trials. YTHDF2 blockade presents a potential paradigm shift in radiosensitization, in that not only are the antitumor effects of radiotherapy enhanced in treated tumors, but also that local radiation can be modified to suppress induction of distant metastasis.

### Limitations of study

First, without in-depth lineage studies, we cannot rule out the possibility that *Ythdf2* knockout in Lyz2 expressing cells may also affect macrophage differentiation and thereby reshape the tumor microenvironment and response to radiation. The exact function of mMDSC-derived subpopulations as well as subtypes of macrophages would require future investigations. Second, the YTHDF2 targets in myeloid cells identified in our work in the context of IR may be restricted to the specific stress condition, or limited by the identification techniques we employed. For instance, IL-10 could be regulated by IR-YTHDF2-NF-κB axis, and might in turn affect NF-κB signaling. The interaction between IL-10 and NF-κB pathway, especially in response to YTHDF2, requires detailed future mechanistic investigation. Third, we designed and synthesized the YTHDF2 inhibitor for our preclinical experiments. We used the current small molecule as a proof-of-principle tool compound to demonstrate antitumor efficacy, when combined with IR, in murine cancer models. Off-target effects may exist and the inhibitor could be further improved in the future. Fourth, although our analysis of the MDSCs and YTHDF2 levels pre- vs. post-RT in patient PBMC is consistent with our conclusions, sample sizes in each trial are relatively small. Further testing with additional clinical samples may provide further support. In spite of these limitations, YTHDF2 inhibition/blockade as a checkpoint inhibitor has the potential to improve radiotherapy or immunotherapy.

## STAR METHODS

### RESOURCE AVAILABILITY

#### Lead contact

Further information and requests for resources and reagents should be directed to and will be fulfilled by the Lead Contact, Ralph R Weichselbaum (rweichselbaum@bsd.uchicago.edu).

#### Materials availability

Request regarding YTHDF2 inhibitor should be addressed to Ralph R Weichselbaum (rweichselbaum@bsd.uchicago.edu), Chuan He (chuanhe@uchicago.edu), and Cheng Luo (cluo@simm.ac.cn).

#### Data availability

The scRNA-seq, RIP-seq, and m^6^A-seq datasets have been deposited in the Gene Expression Omnibus (GEO) under the accession number GSE206387. All deposited data are publicly available as of the date of publication. This study analyzes existing, publicly available ChIP-seq data and the accession number for the dataset is listed in the key resources table.

This paper does not report original code.

Any additional information required to reanalyze the data reported in this paper is available upon request to the lead contact.

### EXPERIMENTAL MODEL AND SUBJECT DETAILS

#### Cells

MC38 and B16 were purchased from ATCC and were maintained according to the method of characterization used by ATCC. LLC cells were obtained from American Type Culture Collection (CRL-1642). B16-OVA were selected as single clones with 5 μg/ml puromycin (InvivoGen) after stable infection with lentivirus-expressing OVA protein. Cells were grown in Dulbecco’s modified Eagle’s medium (DMEM, Gibco) containing 10% heat-inactivated fetal bovine serum (FBS, Gemini), Penicillin (100U/mL)/Streptomycin (100ug/mL, Gibco), and were maintained in a humidified incubator with 5% CO_2_ at 37°C.

#### Mice

All mice were housed and used according to the animal experimental guidelines set by the Institute of Animal Care and Use Committee of The University of Chicago. All animals were maintained in pathogen-free conditions and cared for in accordance with the International Association for Assessment and Accreditation of Laboratory Animal Care policies and certification. *Ythdf2*^flox/flox^ mice were generated using CRISPR-Cas9 technology as described.^79^ *Lyz*^Cre^ mice, *Cd45.1* mice, *Ccr2*^−/−^ mice, *Nfkb1*^−/−^ mice and *Rag1*^−/−^ mice were purchased from The Jackson Laboratory. Male and female mice aged eight to ten weeks were used in the experiments.

#### Patient sample

Patient samples (PBMCs) were obtained from patients treated at the University of Chicago enrolled in the trials NCT02608385^56^ and NCT03223155^57^. Pembro-SBRT study (NCT02608385) and COSINR study (NCT03223155): The studies and amendments were approved by the University of Chicago Biological Sciences Division institutional review board (IRB15-1130 and IRB17-0547, respectively). The studies complied with all ethical regulations and all patients provided written informed consent.

### METHOD DETAILS

#### Tumor growth and treatment

MC38, LLC, B16 or B16-OVA tumor cells were subcutaneously (s.c.) injected in the right flank of mice. For tumor fragment model, MC38 tumors were excised and cut off into fragments, and implanted subcutaneously into recipient mice. Mice were pooled and randomly divided into different groups when the tumor reached a volume of approximately 100 mm^3^ (L × W × H×0.5). The mice were treated with 20 Gy of tumor-localized radiation (one dose) or sham treatment. For anti–PD-L1 treatment experiments, 200 μg of the anti–PD-L1 antibody were injected intraperitoneally twice each week for a total of four times. For YTHDF2 inhibitor treatment, 9 μg of the inhibitor were intravenously injected every day. Tumors were measured twice one week for 3-4 weeks. Animals were euthanized when the tumor volume reached 2, 000 mm^3^ or the diameter of tumor reached 1.5 cm (according to the IACUC protocol). For CD8^+^ T cell depletion experiments, 200 μg of anti-CD8α antibody were delivered by intraperitoneal injection, start from one day before other treatments (twice a week).

#### Flow cytometry

For flow cytometric analysis, tumors, lymph nodes, spleens or blood were collected from mice. The collected tumors tissues were cut into small pieces and were digested with 1 mg/ml collagenase type I or IV (Fisher) and 200 μg/ml DNaseI (Sigma-Aldrich) at 37°C for 60 min to generate the single-cell suspensions. Cells from spleens or lymph nodes were isolated by grinding the tissues through 70 μm filters. Samples were then filtered through a 70 μm cell strainer and washed twice with staining buffer (PBS supplemented with 2% FBS and 0.5 mM EDTA). The cells were re-suspended in staining buffer and were blocked with anti-FcR (2.4G2, BioXcell). Subsequently, the cells were stained with 200-fold diluted fluorescence-labeled antibodies for 30 min at 4°C in the dark and then detected by flow cytometry with a BD Fortessa (BD). For intracellular staining, cells were first permeabilized using a Fixation and Permeabilization Kit (BD) and then stained with appropriate antibodies. Analysis of flow cytometry data was performed using FlowJo V10.

For intracellular staining of YTHDF2, the tumor infiltrating cells were first fixed with Fixation Buffer (BD) for 60 min on ice, and then washed twice with diluted Permeabilization Buffer (BD). Then anti-mouse YTHDF2 antibody (Abcam, ab220163) were added and incubated at 4°C overnight, followed by adding the Alexa Flour 647 goat anti-rabbit IgG (Life technologies) and staining for 60 min.

#### ELISPOT assay

For CD8^+^ T cell functional assay, CD8^+^ T cells were isolated from MC38-OVA tumors, seven days after IR. 2-4 × 10^5^ CD8^+^ T cells were re-stimulated with/without 1 μg/ml SIINFEKEL. After 48-72 hr incubation, the cells were removed. Alternatively, CD11c^+^ DCs were sorted from naïve mice and co-cultured with irradiated tumor cells for 6 hr; then DCs were purified and co-cultured with isolated CD8^+^ T cells for another 48-72 hr. The cytokine spots of IFN-γ were detected with an IFN-γ ELISPOT assay kit according to product protocol. IFN-γ spots were developed according to the manufacturer’s instructions (BD) and calculate by ELISPOT Reader.

#### ELISA

For IL-10 ELISA assay, tumor tissues were collected three days after IR from tumor-bearing WT or *Ythdf2*-cKO mice and were homogenized in PBS with protease inhibitor (1:100). The concentration of IL-10 was measured with an IL-10 Mouse ELISA Kit (Abcam) in accordance with the manufacturer’s instruction.

#### Cytokine detection

For IFN-γ and TNF-α detection, MC38 tumors were collected from WT or *Ythdf2*-cKO mice three days after IR. Tumor tissues were homogenized in PBS with protease inhibitor (1:100), and then centrifuged at 12, 000 rpm for 10 min to collect the supernatant. The supernatant was used to detect the cytokines with LEGENDplex^™^ Mouse Inflammation Panel (13-plex) with V-bottom Plate kit (BioLegend). The samples were detected by flow cytometry with a BD Fortessa (BD). The obtained flow data was analyzed with LEGENDplex software (v8.0, BioLegend).

#### BM-MDSCs induction and isolation

Bone marrow was obtained from wild type, WT or *Ythdf2*-cKO mice and was used to prepare single cell suspension. The cell suspension was called fresh bone marrow cells. The cells were cultured in RPIM-1640 medium containing 10% FBS and 20 ng/ml Recombinant Mouse GM-CSF carrier-free (BioLegend). Fresh medium supplemented with GM-CSF was added on day 3. On day 4, the bone marrow-derived MDSCs (BM-MDSCs) were obtained from fresh bone marrow cells followed with MDSCs isolation using EasySep Selection kits (STEMCELL Technologies).

#### MDSC suppression assay

Murine MDSCs purified from tumors or bone marrow derived MDSCs were performed for the suppression assay. CD8^+^ T cells isolated from the spleen of naïve mice by using EasySep^™^ Mouse CD8^+^ T Cell Isolation Kit (STEMCELL) according to manufacturer’s instructions and then stained with CellTrace CFSE (Invitrogen). The CD8^+^ T cells were cultured with anti-CD3/anti-CD28 beads and were co-cultured with MDSCs at a ratio of 4:1. The CD8^+^ T cells proliferation was analyzed by flow cytometry.

#### Knockdown in BM-MDSCs

SiRNA targeting mouse *Adrb2*, *Metrnl*, or *Smpdl3b* respectively was transfected into bone marrow derived MDSCs by TransIT-TKO^®^ Transfection Reagent (Mirus) according to manufacturer’s protocol. The sequences of siRNA are mouse *Adrb2*: 5’-UAA CAA UCG AUA GCU UUC Utg-3’; mouse *Metrnl*: 5’-UUG AAA GUC ACU AAA GCG Ugg-3’; mouse *Smpdl3b* 5’-UUU GGA UAG GGU GUA GUU Ggg-3’. One-two days after the transfection, the cells were collected. The knockdown efficiency was detected by qPCR.

#### Transwell migration assay

We used 6-well or 24-well transwell plates with 8 μm inserts in polyethylene terephthalate track-etched membranes (Corning). The purified MDSCs from tumors or bone marrow derived cells (5.0×10^6^ cells/insert for 6-well; 1.5×10^6^ cells/insert for 24-well) in serum-free medium were added into the upper compartment of the chamber. The inserts were placed in plates with complete DMEM medium. After incubating overnight, insert membranes were washed with PBS, fixed with 70% methanol for 10 min, and stained with 0.05% crystal violet to detect the migrated cells. An inverted microscope was used for counting.

#### RNA stability assay

MDSCs were sorted from spleen in WT or *Ythdf2*-cKO mice and were seeded in 24-well plates at 50% confluency. 5 μg/mL of Actinomycin D (Sigma-Aldrich) was added. After 0, 0.5, 1, 3, and 6 hours of incubation, cells were collected. The total RNA was purified by RNeasy kit with an additional DNase-I digestion step on the column. RNA quantities were determined using RT-qPCR analysis.

#### Forced expression of YTHDF2 in MDSCs

The cloned *Ythdf2* cDNA with K416A, R527A, W432A, and W486A mutation, which has been proved to significantly decrease the m^6^A binding affinity^80^, were synthesized and cloned into the lentiviral expression vector pLVX-ZsGreen-N1 to generate pLVX-ZsGreen-N1-YTHDF2-Mut (GenScript). The constructed vectors were packaged by co-transfection of 293X cells with two lentiviral helper plasmids pVSVG and pVPR. Virus-containing conditioned medium was harvested 48 h after transfection, filtered, and used to infect BM-MDSCs in the presence of 8 μg/mL polybrene. Infected cells were selected with 2 μg/mL puromycin.

#### RIP-seq

The tumor infiltrated CD11b^+^ myeloid cells were sorted using the EasySep Selection kits (STEMCELL Technologies) from five pooled wild-type (*Ythdf2*^f/f^) mice three days after IR per technical replicate (total three technical replicates). The purified cells were washed with cold PBS and the cell pellet was re-suspended with three packed cell volume of lysis buffer (150 mM KCl, 10 mM HEPES pH 7.5, 2 mM EDTA, 0.5% NP-40, 0.5 mM dithiothreitol (DTT), 1:100 protease inhibitor cocktail, 400 U/ml RNase inhibitor), pipetted up and down several times and incubated on ice for 30 min, and treated with ultrasonic for 1 min. The lysate was centrifuged for 30 min at 1, 4000 rpm (4 °C) to clear the lysate. One-tenth volume of cell lysate was saved as input and mixed with Trizol to extract the total RNA. The rest of the cell lysate was incubated with 20 μg anti-YTHDF2 rabbit polyclonal antibody (Aviva systems biology) at 4 °C overnight with gentle rotation. 200 μL protein G beads were thrice washed with binding buffer, and incubated with cell lysate-antibody mixture at 4 °C for at least 4 h. Then, the protein G beads were collected with magnetic stand, thrice washed with binding buffer, and mixed with Trizol for RNA extraction and saved as IP. Subsequently, the RNA library for sequencing was constructed using SMARTer^®^ Stranded Total RNA-Seq Kit v2 - Pico Input Mammalian (Takara Bio).

#### RIP-qPCR analysis

RIP for YTHDF2 was performed using 20 μg anti-YTHDF2 rabbit polyclonal antibody (Aviva systems biology), as described above. After IP, RNA was isolated from Input and IP fractions using phenol/chloroform extraction. cDNA was prepared with the Applied Biosystems^™^ High-Capacity cDNA Reverse Transcription Kit (Thermo). SYBR-green-based qPCR was performed using QuantiStudio3 (ABI).

#### m^6^A-seq

Total RNA was isolated from tumor infiltrated CD11b^+^ myeloid cells and followed by two rounds of ploy(A) selection to get mRNA. CD11b^+^ myeloid cells were sorted from five pooled *Ythdf2*^f/f^ mice three days after IR per technical replicate (total three technical replicates). The 100ng mRNA was used for m^6^A immunoprecipitation (m^6^A-IP) with the EpiMark *N*^6^-methyladenosine enrichment kit (NEB E1610S) according to the manufacturer’s protocol. The library was constructed using SMARTer^®^ Stranded Total RNA-Seq Kit v2 - Pico Input Mammalian (TaKaRa Bio) and the sequencing was performed at the University of Chicago Genomics Facility on an Illumina NovaSEQ machine in pair-read mode with 100 bp per read.

#### Single cell RNA-seq (scRNA) analysis

Regarding the CD45^+^ immune cells scRNA-seq, single-cell suspensions were obtained from four pooled MC38 tumors in WT mice with or without IR (20 Gy) four days after IR. Samples were stained using Zombie Red^™^ dye (for live cells) for 30 min and then stained for 20 min using an antibody against mouse CD45. Zombine Red^−^CD45^+^ single cells were sorted for library construction of scRNA-seq. Regarding the mMDSCs scRNA-seq, single-cell suspensions were obtained from pooled MC38 tumors in five WT or *Ythdf2*-cKO mice with or without IR (20 Gy) respectively three days after IR. Samples were stained using Zombie Red^™^ dye for 30 min and then stained for 20 min using an antibody against oligo-conjugated antibodies mouse CD45, CD11b, Ly6C respectively (TotalSeq^™^-B). Zombie Red^−^CD45^+^CD11b^+^Ly6C^hi^ single cells were sorted for library construction of scRNA-seq. The library construction was performed at the University of Chicago Genomics Facility, using Chromium Next GEM Single Cell 3’ GEM, Library & Gel Bead Kit v3.1 (Cat: 1000128) purchased from 10x Genomics according to the protocols provided by manufactures. The aimed target cell recovery for each library was 8, 000 and the libraries were sequenced on an Illumina HiSeq X Ten platform at the University of Chicago Genomics Facility.

Raw scRNA-seq data were processed using 10x Genomics Cell Ranger (v6.0.1), including demultiplexing Illumina base call files (BCL) into FASTQ files (with “cellranger mkfastq” function), aligning sequencing reads in FASTQ files to the mouse reference genome (mm10, GENCODE vM23/Ensembl 98 released on July 7, 2020, from 10x Genomics) and counting the unique molecular identifier (UMI) (with “cellranger count” function). As a results, we generated the digital gene expression matrix with the number of UMIs for each gene in each cell.

Low-quality cells were discarded if (1) the number of expressed genes was smaller than 200; (2) the proportion of mitochondrial gene expression were larger than 25%. We further identified and removed potential doublets by using DoubletFinder (v2.0.3) assuming 6% doublet formation rate.^81^ For scRNA-seq of mMDSC, as we applied Cell Hashing method using a series of oligo-tagged antibodies against ubiquitously expressed surface proteins with different barcodes to uniquely label cells from distinct samples, we demultiplex cells into different samples based on HTO enrichment by using HTODemux method in Seurat package (v4.0.6).^82^

The processed whole gene expression matrix was then fed to Seurat (v4.0.6) for downstream analyses.^82^ Briefly, only genes expressed in more than 3 cells were kept, and the UMI count matrix was normalized by using ‘NormalizeData’ function. Later, 2, 000 highly variable genes were identified by using the ‘FindVariableFeatures’ function with the ‘vst’ method, and ‘ScaleData’ function was applied to scale and center the gene expression matrix. Clustering analyses were performed using the first 40 principal components for constructing the shared nearest neighbor (SNN) graph by using ‘FindNeighbors’ function, and then Louvain clustering algorithm was used to group the cells into different clusters. Next, we applied scClassify (v1.2.0)^83^ for cell type classification based on cell types hierarchies constructed from reference datasets (E-MTAB-8832, CD45^+^ immune cells sorted from MC38 tumor-bearing C57BL/6 mice).^84^

#### Bulk RNA-seq analysis

Raw reads were trimmed with Trimmomatic-0.39^85^, then aligned to mouse genome and transcriptome (mm10, version M19, 2018-08-30) using HISAT (version 2.1.0)^86^ with ‘--rna-strandness RF’ parameters. Annotation files (version M19, 2018-08-30, in gtf format for mouse) were downloaded from GENCODE database (https://www.gencodegenes.org/). For mRNA m^6^A-seq, mapped reads were separated by strands with samtools (version 1.9)^87^ and m^6^A peaks on each strand were called using MACS (version 2)^88^ with parameter ‘-nomodel, --keep-dup 5, -g 2.052e9, --tsize 114 -extsize 150’ separately. Significant peaks with q < 0.01 identified by MACS2 were considered. Peaks identified in at least three biological replicates were merged using bedtools (v.2.26.0)^87^ and were used in the following analysis. Reads, from input of m^6^A-seq or YTHFDF2 RIP-seq, on each GENCODE annotated gene were counted using HTSeq^89^ and then differentially expressed genes were called using DESeq2 package in R^90^ requiring at least 10 read counts in at least three samples with adjusted *p*-value < 0.05. YTHDF2 target genes were identified as differentially up-regulated genes comparing YTHDF2 IP sample with the corresponding Input samples. Functional enrichment analysis was performed with DAVID^91^.

#### TCGA data analysis

TCGA data was acquired and analyzed in part using the Xena Platform.^92^ For survival analysis an MDSC signature score based on expression of *ARG1, CD14, CD44, CD40, S100A8, SELPLG, STAT6, TFRC, TGFB2, STAT3, CD274, ITGA3, SLA*, and *KDR* was applied to the TCGA data. For the Low Grade Glioma (LGG) and Glioblastoma (GBM) cohorts, gene expression was divided into the highest and lowest thirds. Samples in the lowest and highest groups received a score of −1 and 1, respectively. Scores were then added for each sample and samples with a final score within the highest or lowest third was given a ‘High’ or ‘Low’ MDSC signature score, respectively. For all cohorts samples were filtered by ‘Primary Tumor’ and for patients receiving radiation therapy. Survival data from these groups were then compared and significance calculated using the ggsurvplot function in R.

#### Chromatin immunoprecipitation (ChIP) assay

ChIP assays were conducted with a Magna ChIP^™^ A/G Chromatin Immunoprecipitation Kit (Sigma/Millipore). Briefly, 5-10 × 10^6^ BM-MDSCs were fixed with a final concentration of 1% formaldehyde, cross-linked, and sonicated. The anti-RELA antibody (10 μg/mL, CST), or IgG control antibody was added to sonicated lysates and incubated overnight at 4°C, then incubated with Protein A/G beads mixture (1:1 at ratio) for another > 7 h at 4°C. Chromatin DNA was eluted, reverse cross-linked, and recovered using a QIAquick Extraction Kit (Qiagen). Input DNA and immunoprecipitated DNA were analyzed by quantitative PCR using the *Ythdf2* promoter DNA-specific primers.

#### Chemistry

##### Synthesis of 5-(3-hydroxyphenyl)thiophene-2-carbaldehyde

Add the hydroxyphenylboronic acid (63 mg, 0.46 mmol) and 5-bromothiophene-2-carbaldehyde (105 mg, 0.5 mmol) and Pd(DPPF)Cl2.CH2Cl2 (5 mol %) to a mixture of DME/2M Na2CO3 (8 mL, 3/1, v/v) under Ar, then stir the reaction at 80 °C during 6 h. After reaction completed, add 10 mL water to the reaction and extract the aqueous solution with ethyl acetate, combined organic phases were dried over by MgSO4 and the crude residue was purified by flash column chromatography (silica gel, EtOAc/PE) to obtain the product, yellow solid, yield 51.6%. LCMS (ESI) Calcd for C11H8O2S [M+H]+ 204.02, found 204.24. 1H NMR (400 MHz, DMSO) δ 9.90 (s, 1H), 9.79 (s, 1H), 8.02 (d, J = 4.0 Hz, 1H), 7.66 (d, J = 4.0 Hz, 1H), 7.28 (t, J = 7.8 Hz, 1H), 7.24 – 7.20 (m, 1H), 7.14 (t, J = 2.1 Hz, 1H), 6.88 – 6.81 (m, 1H).

##### Synthesis of (E)-2-cyano-3-(5-(3-hydroxyphenyl)thiophen-2-yl)acrylamide

Stir 5-(3-hydroxyphenyl)thiophene-2-carbaldehyde (48 mg, 0.24 mmol), 2-cyanoacetamide (0.29 mmol) and piperdine (0.24 mmol) in ethanol (10 mL) at room temperature overnight. Concentrated the reaction mixture to obtain the crude residue and purified the residue by reverse phase preparative HPLC (CH3CN/water with 0.1% TFA) to obtain yellow solid, yield 64.5%. HRMS (ESI) Calcd for C14H10N2O2S [M+H]+ 271.0536, found 271.0533. 1H NMR (500 MHz, DMSO) δ 9.75 (s, 1H), 8.36 (s, 1H), 7.89 – 7.62 (m, 4H), 7.29 (t, J = 7.7 Hz, 1H), 7.22 (d, J = 7.6 Hz, 1H), 7.15 (s, 1H), 6.83 (d, J = 8.1 Hz, 1H). 13C NMR (126 MHz, DMSO) δ 163.1, 158.5, 151.9, 143.9, 140.0, 135.1, 134.0, 131.0, 125.3, 117.4, 117.3, 1171, 113.1, 101.8.

#### Protein Expression and Purification

YTHDF2 (aa 380-559) and YTHDF1 (aa 361-559) were first cloned into modified pET28a-TEV vector. The plasmid was transformed into E. coli BL21 (DE3) cells and the proteins were induced with 1 mM Isopropyl- -D-thiogalactopyranoside (IPTG) for 16 hours at 20°C. The cells were collected and resuspended in the lysis buffer containing 20 mM Tris (pH 7.4), 150 mM NaCl, 0.05% (v/v) - mercaptoethanol and 5% (v/v) glycerol. YTHDF2 (aa 380-579) and YTHDF1 (aa 361-559) were then purified through Ni-NTA chromatography (HisTrap FF, GE Healthcare), followed by the purifications including a cation exchange column and a Superdex 75 10/300 column. The purified proteins were stored at −80°C in the buffer containing 20 mM Hepes (pH 7.4) and 200 mM NaCl.

#### Fluorescence Polarization (FP)

The high throughput screening (HTS) of the laboratory’s in-house compound library was performed at the final concentration of 80 μM. Diluted compounds were first incubated with 1.25 μM YTHDF2 (aa 380-579) for half an hour at 25°C in the binding buffer containing 20 mM Hepes (pH 7.4), 50 mM NaCl, 5% (v/v) glycerol and 0.01% (v/v) tween 20. And 30 nM fluorescently-labeled m6A-containing mRNA (5’-FAM-UUCUUCUGUGG (m6A) CUGUG-3’) was then added and incubated for another one hour at 4°C before testing via Envision Readers (PerkinElmer). The same amount of DMSO was used as the negative control, unlabeled m^6^A-containing mRNA with the same sequence was used as the positive control and the 5’-FAM-labeled m^6^A-containing mRNA was utilized to ascertain the gain factor.

#### AlphaScreen

The compound DC-Y13 and DC-Y13-27 were first diluted from 1 mM to concentrations as indicated using double dilution method, respectively. Then His-tagged YTHDF2 (aa 380-579) or His-tagged YTHDF1 (aa 361-559) was added to the diluted compounds at the final concentration of 80 nM. The same amount of DMSO and the unlabeled m^6^A-containing mRNA were separately served as the negative control and the positive control, respectively. The samples were incubated in the binding buffer containing 20 mM Hepes (pH 7.4), 150 mM NaCl, 0.01% (v/v) TritonX-100 and 1 mg/ml BSA for half an hour at 25°C before biotinylated m^6^A-containing mRNA (5’-biotin-UUCUUCUGUGG (m^6^A) CUGUG-3’) was added at the final concentration of 10 nM. Next, the mixture of anti-His acceptor beads and streptavidin donor beads were added away from light. And the samples were then incubated for another one hour at 4°C and then measured on Envision Readers (PerkinElmer).

#### Microscale Thermophoresis (MST)

The compound DC-Y13-27 was first diluted from 2.5 mM using a double dilution method with the MST assay buffer containing 20 mM Hepes (pH 7.4), 200 mM NaCl and 0.1% (v/v) Pluronic^®^ F-127. Then, the compound samples were incubated with YTHDF2 (aa 380-579) at the final concentration of 2 μM for 20 minutes at 25°C followed by 10 minutes of 13, 000 rpm centrifugation at 4°C before the detection. Next, prepared samples were loaded into the Monolith NT. Automated LabelFree Premium Capillary Chips (NanoTemper Technologies) and the experiments were performed using the label-free method on the Monolith NT. Automated instrument (NanoTemper Technologies). The binding constant (KD) of DC-Y13-27 and YTHDF2 (aa 380-579) was acquired by analyzing data using the MO. Affinity Analysis Software v2.3 (NanoTemper Technologies).

#### Method-Surface Plasmon Resonance Assay

Surface plasmon resonance assay was performed to test the binding affinity of YTHDF2 protein and DC-Y13-27 on a Biacore T200 instrument (GE Healthcare) with HBS buffer (20 mM HEPES pH7.4, 200 mM NaCl, 0.08% (v/v) DMSO) at 25°C. The protein was immobilized on a CM5 chip (GE Healthcare) using a standard amine-coupling procedure in 10 mM sodium acetate (pH 4.0). The chip was then equilibrated in HBS buffer. The compound was serially dissolved with HBS buffer. For each cycle, compound solution was injected for 120 s, followed by a 300 s delay for dissociation. The KD values were determined by Biacore T200 evaluation software (GE Healthcare).

### QUANTIFICATION AND STATISTICAL ANALYSIS

To estimate the statistical significance of differences between two groups, we used a paired or unpaired Student’s *t*-tests to calculate two-tailed P values. One-way analysis of variance (ANOVA) or two-way ANOVA with multiple comparison test was performed when more than two groups were compared. Survival analysis was performed using Kaplan-Meier curves and evaluated with log-rank Mantel-Cox tests. Error bars indicate the standard error of the mean (SEM) unless otherwise noted. P values are labeled in the figures. P values were denoted as follows: *P< 0.05, ** *P*< 0.01, *** *P*< 0.001, **** *P*< 0.0001. Statistical analyses were performed by using GraphPad Prism (version 8.0).

## Supplementary Material

1 Table S1. Signature genes in each cell population in tumors, related to Figure 3.

2 Table S2. Signature genes in each combined cell population, related to Figure 3

3

## Figures and Tables

**Scheme 1.**
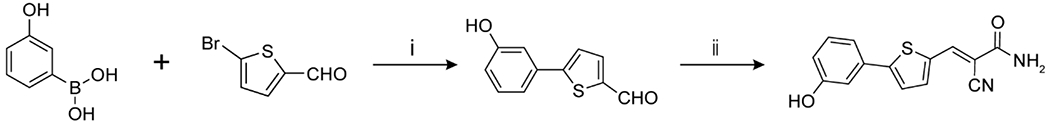
The design and synthesis of compound DC-Y13-27. Conditions and reagents: i) Pd(DPPF)Cl2.CH2Cl2, Na2CO3, 80 °C; ii) piperdine, EtOH, rt.

**Table:** Key resources table

REAGENT or RESOURCE	SOURCE	IDENTIFIER
Antibodies		
Anti-CD45	BioLegend	Cat# 103116, RRID:AB_312981
Anti-CD8a	BioLegend	Cat# 100705, RRID:AB_312744
Anti-CD8a	BioLegend	Cat# 100708, RRID:AB_312747
Anti-CD45.2	BioLegend	Cat# 109828, RRID:AB_893350
Anti-CD45.1	BioLegend	Cat# 110714, RRID:AB_313503
Anti-Ly-6C	BioLegend	Cat# 128006, RRID:AB_1186135
Anti-CD11b	BioLegend	Cat# 101224, RRID:AB_755986
Anti-CD11b	BioLegend	Cat# 101208, RRID:AB_312791
Anti-IFN-gamma	BioLegend	Cat# 505809, RRID:AB_315403
Anti-CD11c	BioLegend	Cat# 117310, RRID:AB_313779
Anti-CD11c	BioLegend	Cat# 117306, RRID:AB_313775
Anti-I-A/I-E	BioLegend	Cat# 107620, RRID:AB_493527
Anti-Ly-6G	BioLegend	Cat# 127622, RRID:AB_10643269
Anti-Ly-6G	BioLegend	Cat# 127608, RRID:AB-1186099
Anti-F4/80	BioLegend	Cat# 123108, RRID:AB_893502
Anti-CD4	BioLegend	Cat# 100406, RRID:AB_312691
Anti-CD4	BioLegend	Cat# 100407, RRID:AB_312692
Anti-CCR2	R&D Systems	Cat# FAB5538A, RRID:AB_10645617
Anti-YTHDF2	Aviva Systems Biology	Cat# ARP67917_P050, RRID:AB_2861185
Anti-YTHDF2	Abcam	Cat# ab220163; RRID:AB_2868573
Anti-Histone H3	Abcam	Cat# ab201456, RRID:AB_2650560
Anti-NF-κB p65	Cell Signaling Technology	Cat# 4764S
Anti-IκBα (L35A5)	Cell Signaling Technology	Cat# 4814S
Anti-Phospho-IκBα	Cell Signaling Technology	Cat# 2859S
Anti-CCR2	R&D Systems	Cat# FAB5538A, RRID:AB_10645617
Anti-β-Actin	Santa Cruz Biotechnology	Cat# sc-47778, RRID:AB_626632
Donkey anti-rabbit IgG-HRP Polyclonal	Santa Cruz Biotechnology	Cat# sc-2313, RRID:AB_641181
Goat anti-mouse IgG-HRP Polyclonal	Santa Cruz Biotechnology	Cat# sc-2005, RRID:AB_631736
Goat anti-Rat IgG (H+L) Cross-Adsorbed Secondary Antibody, Alexa Fluor^™^ 647	Thermo Fisher	Cat# A-21247, RRID:AB_141778
Fc Block	Bio X Cell	Cat# BE0307, RRID:AB_2736987
InVivoMAb anti-mouse CD8α	Bio X Cell	Cat# BE0004-1, RRID:AB_1107671
InVivoMAb anti-mouse PD-L1 (B7-H1)	Bio X Cell	Cat# BE0361
		
Biological samples		
Patient PBMCs	University of Chicago	trials NCT02608385 and NCT03223155
		
Chemicals, peptides, and recombinant proteins		
Collagenase, Type I	Fisher Scientific	Cat# LS004197
DNase I	Sigma	Cat# 10104159001
Recombinant Mouse GM-CSF	BioLegend	Cat# 576306
Recombinant Mouse IL-2 (carrier-free)	BioLegend	Cat# 575406
Dynabeads^™^ Mouse T-Activator CD3/CD28	Thermo Fisher	Cat# 11453D
Cell Stimulation Cocktail	Thermo Fisher	Cat# 00-4970-93
Actinomycin D	Sigma	Cat# A9415-5MG
Absolute Counting Beads	Invitrogen	Cat# C36950
Protease inhibitor	Thermo Fisher	Cat# 1861278
TransIT-TKO^®^ Transfection Reagent	Mirus	Cat# MIR2150
BAY 11-7082	Fisher	Cat# HY-13453
		
Critical commercial assays		
Mouse IFN-gamma ELISPOT set	Fisher Scientific	Cat# BDB551083
Magna RIP^™^ RNA-Binding Protein Immunoprecipitation Kit	Sigma	Cat# 17-700
Dynabeads^™^ mRNA DIRECT^™^ Purification Kit	Invitrogen	Cat# 61011
EasySep^™^ Mouse CD8+ T Cell Isolation Kit	STEMCELL	Cat# 19853
EasySep^™^ Mouse Monocyte Isolation Kit	STEMCELL	Cat# 19861
LEGENDplex^™^ Mouse Inflammation Panel (13-plex)	BioLegend	Cat# 740446
Mouse IL-10 Quantikine ELISA Kit	R&D Systems	Cat# M1000B
Fixation and Permeabilization Solution	BD Bioscience	Cat# 554722
CFSE Cell Proliferation Kit	Invitrogen	Cat# C34554
SMARTer^®^ Stranded Total RNA-Seq Kit v2 - Pico Input Mammalian	TaKaRa	Cat# 634413
Mouse IFN-γ Flex Set	BD Bioscience	Cat# 558296
Magna ChIP^™^ A/G Chromatin Immunoprecipitation Kit	Sigma	Cat# 17-10085
Power SYBR^™^ Green PCR Master Mix	Thermo Fisher	Cat# 4367659
High-Capacity cDNA Reverse Transcription Kit	Thermo Fisher	Cat# 4374966
N6-Methyladenosine Enrichment Kit	NEB	Cat# E1610S
TotalSeqtrade-B0301 anti-mouse Hashtag 1	BioLegend	Cat# 155831
TotalSeqtrade-B0302 anti-mouse Hashtag 2	BioLegend	Cat# 155833
		
		
Deposited data		
Data files for scRNA-seq, RIP-seq, and m6A-seq	This paper	GEO: GSE206387
Bone marrow-derived macrophages ChIP-seq	Nguyen et al.^78^	GEO: GSE99895
		
Experimental models: Cell lines		
MC38	ATCC	CRL-2640
B16F1	ATCC	CRL-6323
B16-OVA	Maintained in our Lab	N/A
LLC	ATCC	CRL-1642
		
Experimental models: Organisms/strains		
C57BL/6 mice	Harlan - Envigo	N/A
*Lyz*^cre^ mice	JAX	N/A
*Ythdf2*^flox/flox^ mice	Li et al.^79^	N/A
*Nfkb1* KO mice	JAX	N/A
*Rag1* KO mice	JAX	N/A
*Ccr2* KO mice	JAX	N/A
*CD45.1* mice	JAX	N/A
		
Recombinant DNA		
pLVX-ZsGreen-N1-Ythdf2 mut	GenScript	Cat# SC 1692
mutated Ythdf2 cDNA	GenScript	Cat# SC1010
		
Software and algorithms		
GraphPad Prism	GraphPad	https://www.graphpad.com/scientific-software/prism/
FlowJo V10	FlowJo	https://www.flowjo.com/solutions/flowjo
Seurat package (v4.0.6)	Stuart et al.^82^	https://github.com/satijalab/seurat/releases/tag/v4.0.6
scClassify (v1.2.0)	Lin et al.^83^	https://github.com/SydneyBioX/scClassify_analysis
Trimmomatic-0.39	Bolger, et al.^85^	http://www.usadellab.org/cms/index.php?page=trimmomatic
HISAT (version 2.1.0)	Kim et al.^86^	https://github.com/DaehwanKimLab/hisat2
MACS (version 2)	Zhang et al.^88^	http://liulab.dfci.harvard.edu/MACS/
samtools (version 1.9)	Danecek et al.^87^	http://www.htslib.org/
bedtools (v.2.26.0)	Danecek et al.^87^	http://www.htslib.org/
HTSeq	Anders et al.^89^	http://www-huber.embl.de/HTSeq

## Highlights

- YTHDF2 elevation in myeloid cells post-RT correlates with poor outcome in patients
- YTHDF2 depletion or inhibition in myeloid cells augments antitumor immunity of IR
- YTHDF2 depletion alters MDSC sub-populations in blood and tumors after IR treatment
- The YTHDF2-NF-κB circuit regulates MDSC migration and suppressive function
