# Supplementary material for: YTHDF2 inhibition potentiates radiotherapy anti-tumor efficacy
Source: Cancer Cell. Author manuscript; Available in PMC 2024 Jul 10. (PMC10524856; doi:10.1016/j.ccell.2023.04.019)
Supplement: 3 [file NIHMS1899599-supplement-3.pdf]

## Supplemental information Figures and Legends

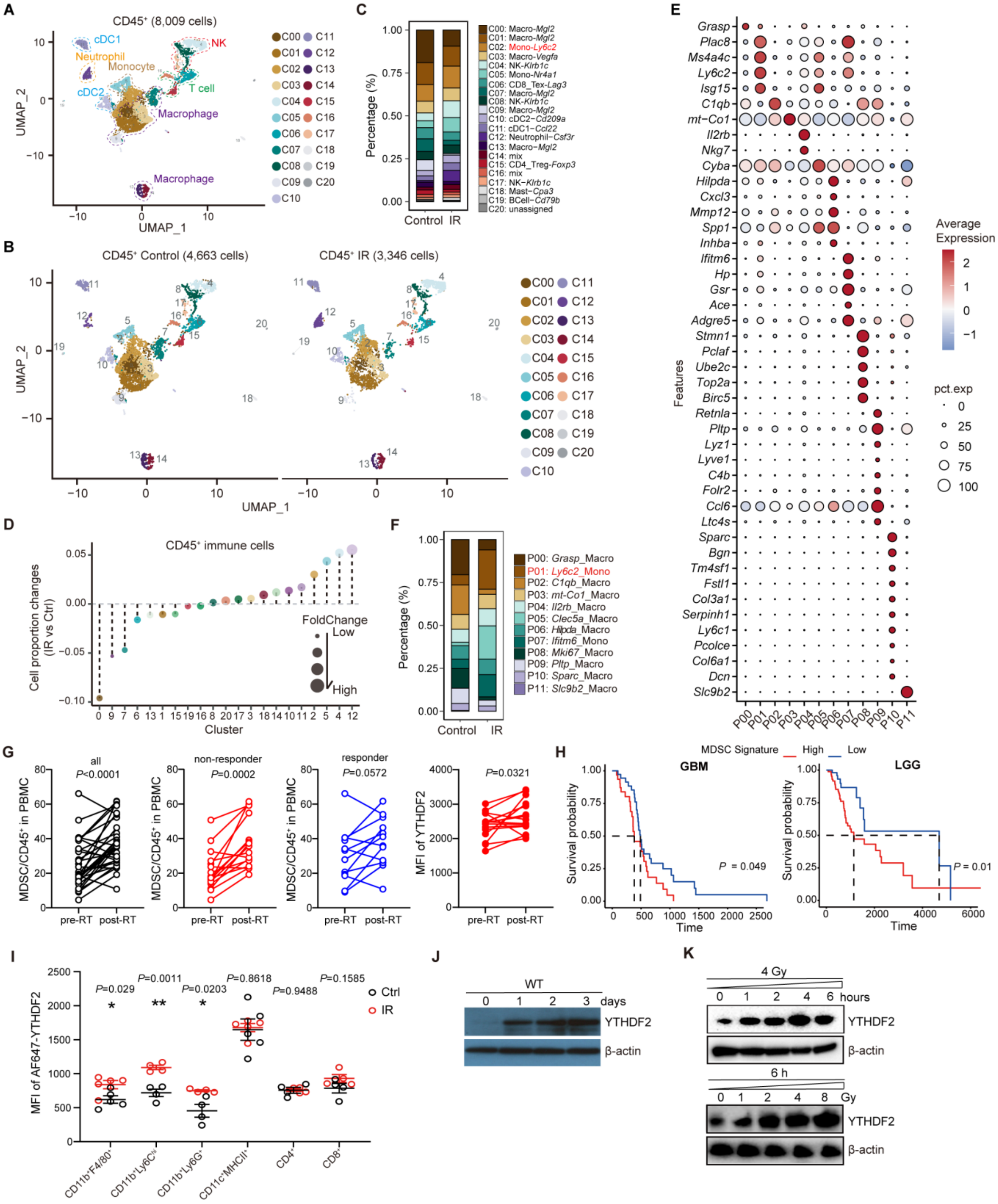

**Figure S1. scRNA-seq identified CD45$^{+}$ immune cell populations and YTHDF2 expression in IR-treated MDSCs. Related to Figure 1.**

**(A-B)** UMAP showing five clusters including T cells, NK, Macrophages, DCs and Monocytes (A) from scRNA-seq using sorted $CD45^+$ immune cells from MC38 tumors with and without IR treatment, respectively (B) four days after IR.

**(C)** Proportion of different cell subsets of $CD45^+$ immune cells (as in Figure S1A-B) in irradiated vs. non-irradiated MC38 tumors.

**(D)** Changes in proportion of different cell subsets of $CD45^+$ immune cells (as in Figure S1C) in irradiated vs. non-irradiated MC38 tumors.

**(E)** Bubble heatmap showing the expression of feature genes of each myeloid cells cluster from Figure 1A.

**(F)** Proportion of different cell subsets of myeloid cells (as in Figure 1A) in irradiated vs. non-irradiated MC38 tumors.

**(G)** Flow cytometry analysis of MDSCs in PBMCs from metastatic NSCLC patients enrolled in a clinical trial (the COSINR study, NCT03223155) (pre-RT vs. post-RT) and Mean Fluorescent Intensity (MFI) of YTHDF2 in MDSCs of PBMC from non-responders patients. Non-responder was characterized as < 8 month to 'progression or death' (the average progression time), while responder was characterized as with > 8 months to 'progression or death'.

**(H)** Overall survival analysis of cancer patients in Low Grade Glioma (LGG), and Glioblastoma (GBM) cohorts and either high or low MDSC signature. Normalized gene expression and corresponding clinical data on patients were obtained from TCGA.

**(I)** Flow cytometric analysis of YTHDF2 expression in different immune cells (as indicated) isolated from irradiated MC38 tumors. Macrophages: $CD11b^+F4/80^+$; mMDSC: $CD11b^+Ly6C^{hi}$ $Ly6G^-$; PMN-

MDSC: $CD11b^+Ly6G^+$ $Ly6C^-$; DCs: $CD11c^+MHCII^+$; $CD4^+$ T: $CD45^+CD4^+$; $CD8^+$ T: $CD45^+CD8a^+$; (n = 4-5 per group)

**(J)** MDSCs sorted from tumors in MC38-bearing WT mice one, two, and three days after IR. Immunoblot analysis of YTHDF2 in sorted MDSCs.

**(K)** MDSCs were derived from bone marrow cells (WT mice) and treated with different doses of IR directly and cultured for different times as indicated. Graph showing the immunoblot analysis of YTHDF2.

Data are represented as mean ± s.e.m., Statistical analysis was performed using two-sided paired Student's *t*-test (G) or two-sided unpaired Student's *t*-test (I). *$P < 0.05$ and **$P < 0.01$.

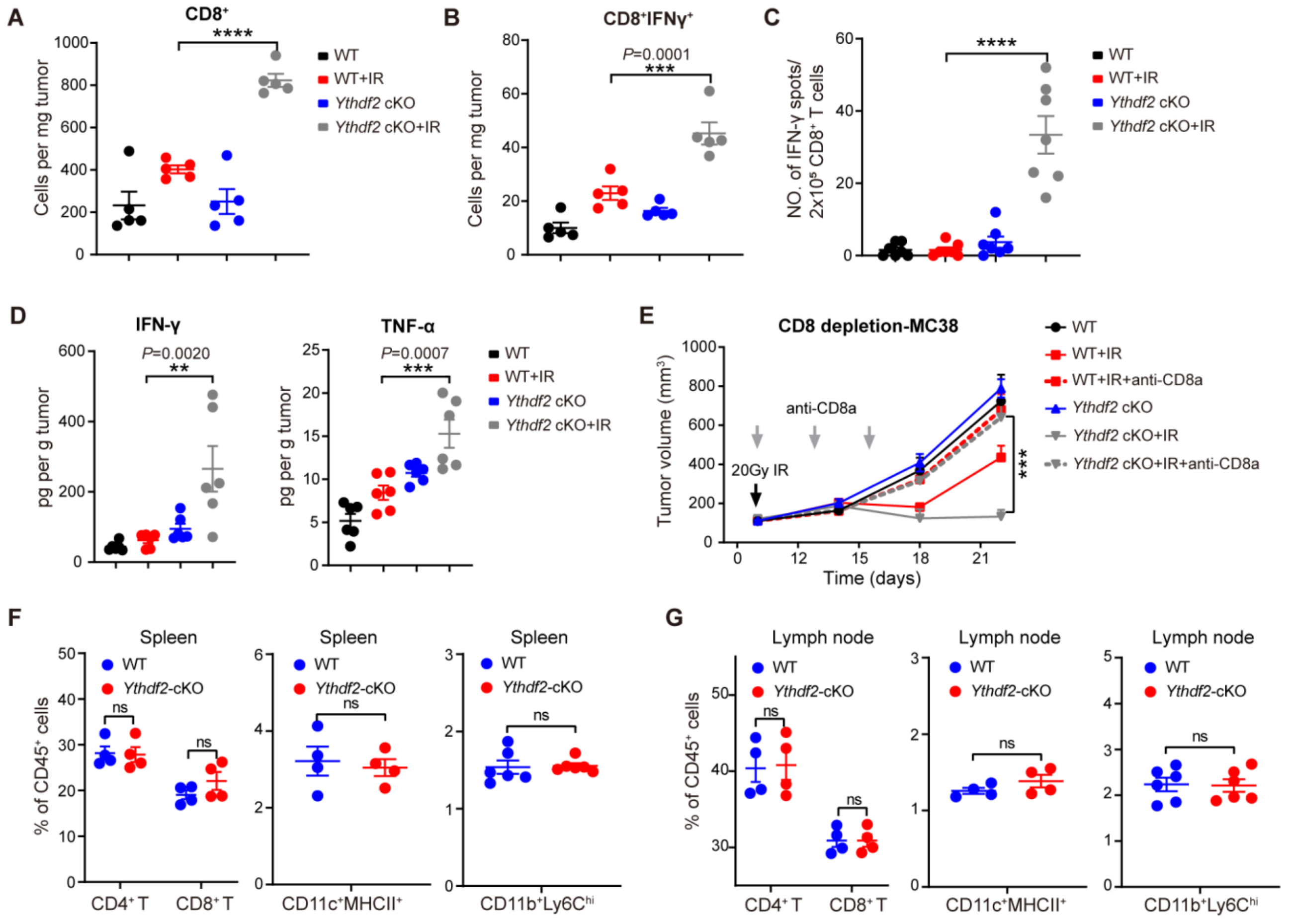


**Figure S2. The antitumor effects of IR in *Ythdf2*-cKO mice depend on CD8$^+$ T cell response. Related to Figure 2.**

**(A-B)** The numbers of MC38 tumor-infiltrating total CD8$^+$ T cells (**A**) and CD8$^+$IFNγ$^+$ T cells (**B**) in WT or *Ythdf2*-cKO mice with/without IR (7 days after IR). (n = 5 per group)

**(C)** The CD8$^+$ T cells were isolated from MC38 tumors (in A) and the IFN-γ spots were enumerated by ELISPOT assay.

**(D)** The MC38 tumor tissues (in A) were collected to measure the levels of IFN-γ and TNF-α using the LEGEND plex cytokine kit.

**(E)** WT or *Ythdf2*-cKO mice were injected subcutaneously with 1x10$^6$ MC38 cells. When the tumor size reached 100 mm$^3$, mice were treated with 200 μg of CD8a-depleting antibody twice a week

starting on the same day of tumor-local IR (20 Gy, one dose). Tumor growth was monitored. (n = 5 per group)

**(F-G)** Percentages of $CD4^+$, $CD8^+$, DCs ($CD11c^+MHCII^+$) and mMDSCs ($CD11b^+Ly6C^{hi}$ $Ly6G^-$) in Spleen (F) and lymph node (G) in WT and *Ythdf2*-cKO mice. (n = 4-6 per group)

Data are represented as mean ± s.e.m., n, number of mice. One of two representative experiments was shown. Statistical analysis was performed using one-way ANOVA with Bonferroni's multiple comparison tests (A-D), two-way ANOVA test with corrections for multiple variables (E), or two-sided unpaired Student's *t*-test (F, G). $*P < 0.05$, $**P < 0.01$, and $***P < 0.001$.

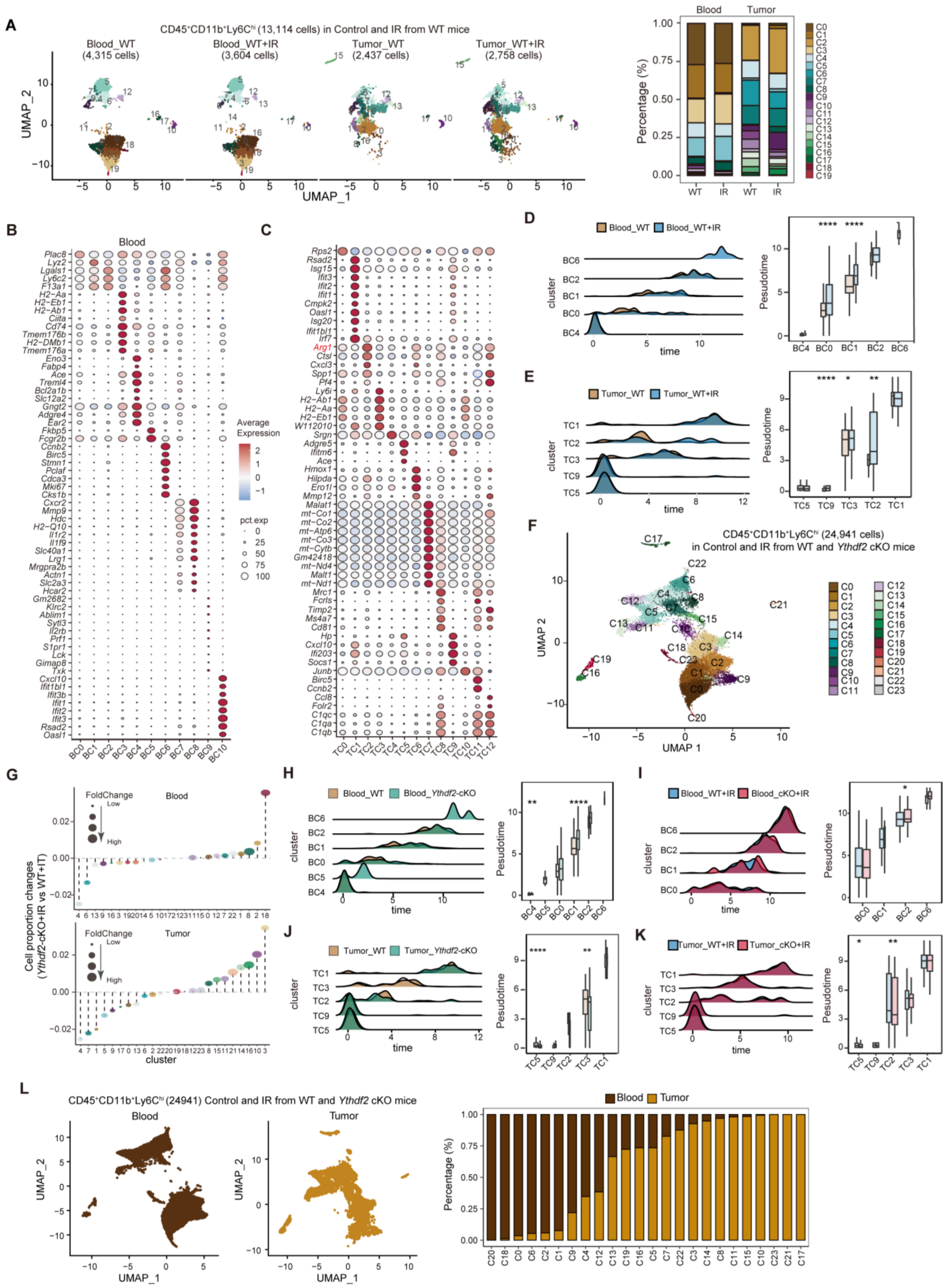

**Figure S3. IR or YTHDF2 affects mMDSC differentiation in both blood and tumors. Related to Figure 3.**

**(A)** UMAP plot displaying different mMDSC-derived subsets from blood and tumor with/without IR based on the scRNA-seq data of tumor-infiltrating $CD45^+CD11b^+Ly6C^{hi}$ cells (left). Proportion of different mMDSC-derived subsets (right). The cells were sorted from blood or tumors in five pooled IR-treated MC38 tumor-bearing mice three days after IR.

**(B)** Bubble heatmap showing the expression of feature genes of each mMDSC-derived cluster in blood from **Fig. 3C**.

**(C)** Bubble heatmap showing the expression of feature genes of each mMDSC-derived cluster in tumor from **Fig. 3D**.

**(D)** Density plot (left) and boxplot (right) showing pseudotime of cells within each cluster in blood with or without IR treatment.

**(E)** Density plot (left) and boxplot (right) showing pseudotime of cells within each cluster in tumors with or without IR treatment.

**(F)** Cell population in blood and tumors from WT or *Ythdf2*-cKO mice with/without IR.

**(G)** Proportion of mMDSC-derived subsets (from **Fig. 3F**) in blood and tumors from WT and *Ythdf2*-cKO mice with non-IR versus IR treatment.

**(H)** Density plot (left) and boxplot (right) showing pseudotime of cells within each cluster in blood from WT and *Ythdf2*-cKO mice without IR treatment.

**(I)** Density plot (left) and boxplot (right) showing pseudotime of cells within each cluster in blood from WT and *Ythdf2*-cKO mice with IR treatment.

**(J)** Density plot (left) and boxplot (right) showing pseudotime of cells within each cluster in tumors from WT and *Ythdf2*-cKO mice without IR treatment.

**(K)** Density plot (left) and boxplot (right) showing pseudotime of cells within each cluster in tumors from WT and *Ythdf2*-cKO mice with IR treatment.

(**L**) (Left) UMAP plot displaying $CD45^{+}CD11b^{+}Ly6C^{hi}$ cells in blood and tumors from WT or *Ythdf2*-cKO mice with/without IR. Cells were colored according to which tissue they belong to. (Right) A barplot illustrating the ratio of the number of cells from blood to the number of cells from tumor in each cluster. The clusters were ranked based on the ratio.

For box plots (D, E, H-K), the center line represents the median, the box limits show the upper and lower quartiles, whiskers represent 1.5x the interquartile range. *P* values were calculated by a nonparametric Wilcoxon-Mann-Whitney test. * $P < 0.05$, ** $P < 0.01$, *** $P < 0.001$, **** $P < 0.0001$.

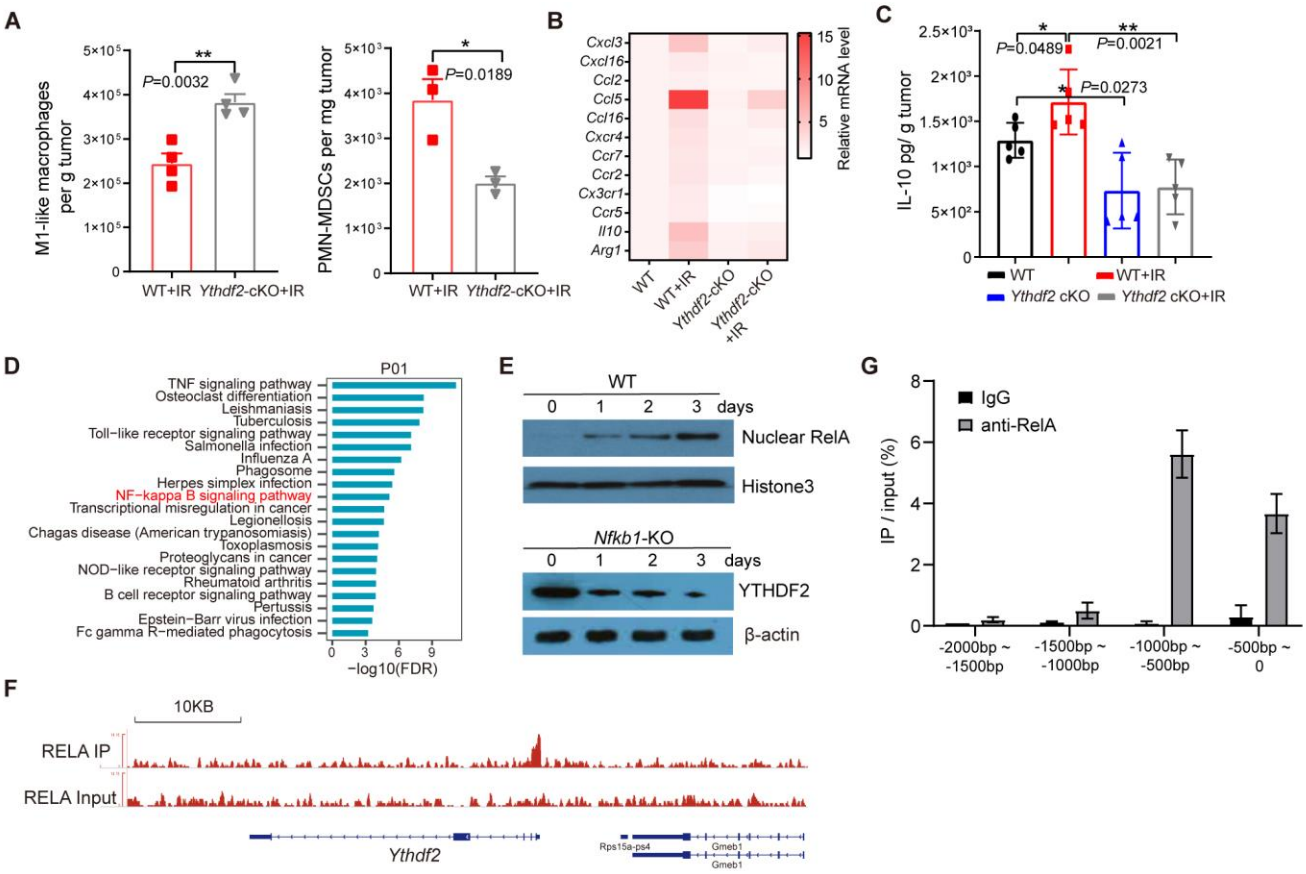


**Figure S4. *Ythdf2* deletion inhibits MDSC suppression function and NF-κB/RELA mediates IR-induced YTHDF2 expression in MDSCs. Related to Figures 3 and 4.**

**(A)** Percentages of MC38 tumor-infiltrating M1-like macrophages (CD45$^+$CD11b$^+$MHCII$^+$CD206$^-$; left) and PMN-MDSCs (CD45$^+$CD11b$^+$Ly6G$^+$Ly6C$^-$; right) assessed by flow cytometry (treatment conditions as indicated). (n = 3-4 per group)

**(B)** MDSCs were sorted from MC38 tumors in WT, WT+IR, *Ythdf2*-cKO, *Ythdf2*-cKO+IR mice three days after IR and subjected to qPCR analysis of the mRNA level of genes as indicated. (n = 3 per group)

**(C)** The MC38 tumor tissues were collected in WT, WT+IR, *Ythdf2*-cKO and *Ythdf2*-cKO+IR mice to measure the protein level of IL-10 by ELISA. (n = 5 per group)

**(D)** Function enrichment analysis of gene signatures of *Ly6c2*_monocytes (P01) cluster in Fig.1A.

**(E)** Immunoblot analysis of nuclear RELA in sorted MDSCs from tumors in MC38-bearing WT mice one, two, and three days after IR (top). Immunoblot analysis of YTHDF2 in sorted MDSCs from tumors in MC38-bearing *Nfkb1* knockout mice one, two, and three days after IR (bottom).

**(F)** Profile of RELA binding (GSE99895) at the promoter region of *Ythdf2* in bone marrow-derived macrophages.

**(G)** Chromatin immunoprecipitation (ChIP) analysis of the *Ythdf2* promoter in BM-MDSCs.

Data are represented as mean ± s.e.m., n, number of mice. Statistical analysis was performed using two-tailed unpaired Student's *t* test (A), one-way ANOVA with Bonferroni's multiple comparison tests (C). $^{*}P < 0.05$, $^{**}P < 0.01$.

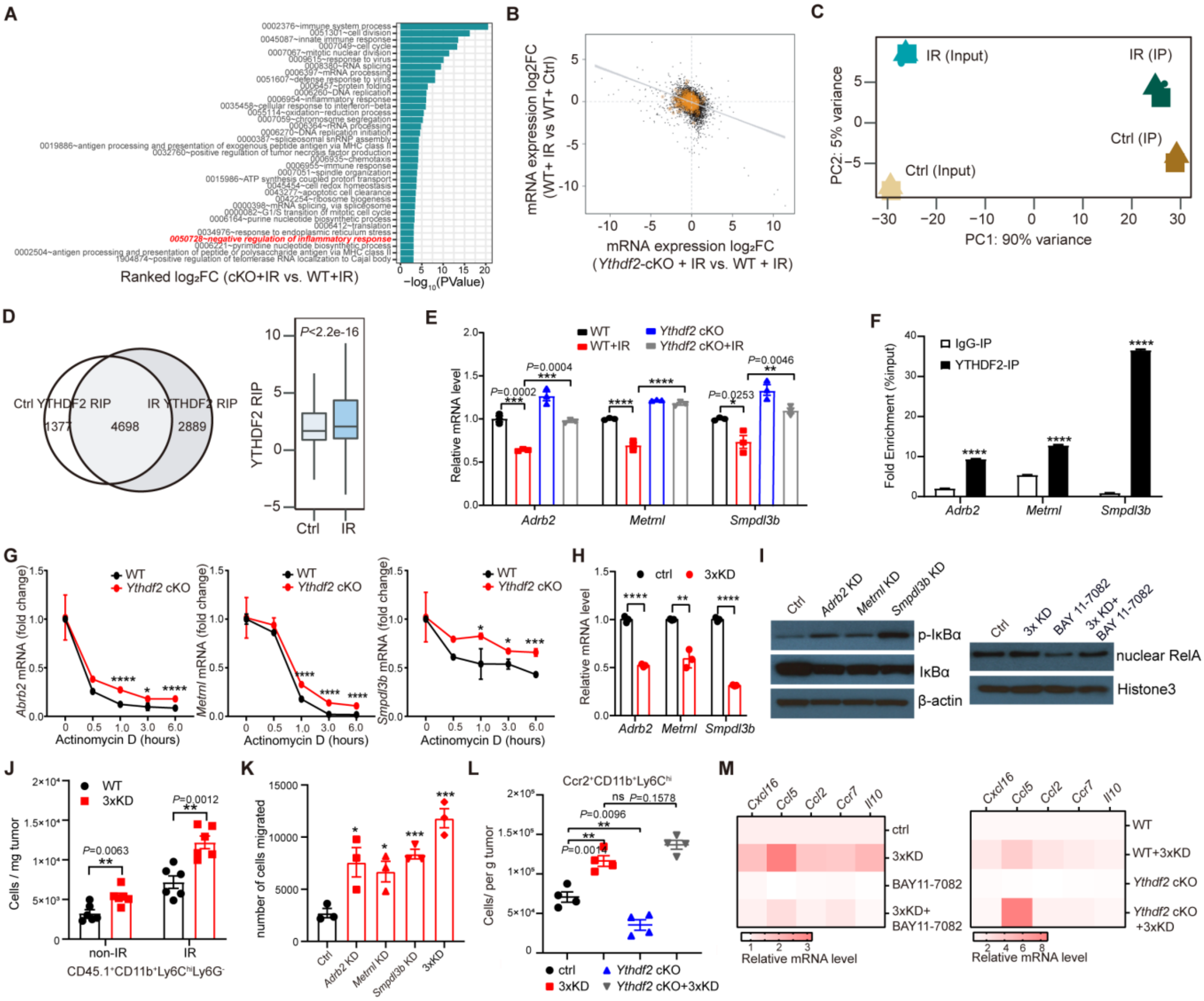

**Figure S5. YTHDF2 degrades *Adrb2*, *Metrnl*, and *Smpdl3b* transcripts and thereby activates NF-κB signaling in MDSCs. Related to Figure 5.**

**(A)** Functional enrichment analysis of genes in tumor-infiltrating CD11b$^+$ myeloid cells that were downregulated upon WT+IR vs. WT, and also upregulated upon *Ythdf2*-cKO+IR vs. WT+IR using DAVID.

**(B)** Scatter plot of gene expression fold changes (log2FC) between IR versus Control, and *Ythdf2*-cKO+IR versus WT+IR in CD11b$^+$ myeloid cells.

**(C)** PCA analysis of YTHDF2 RIP-seq (as in Figure 5E).

**(D)** Venn diagram of YTHDF2 targets (from YTHDF2 RIP-seq data) in Control and IR condition (left). Boxplot showing YTHDF2 binding intensity on its target genes in Control and IR condition (right). For box plots, the center line represents the median, the box limits show the upper and lower quartiles, whiskers represent 1.5x the interquartile range. *P* values were calculated by a nonparametric Wilcoxon-Mann-Whitney test.

**(E)** MDSCs were sorted from MC38 tumors in WT, WT+IR, *Ythdf2*-cKO, *Ythdf2*-cKO+IR mice and subjected to qPCR analysis of *Adrb2*, *Metrnl* and *Smpdl3b* mRNA level. (n = 3 per group)

**(F)** Graphs showing enrichment of *Adrb2*, *Metrnl* and *Smpdl3b* mRNA in the YTHDF2-immunoprecipitated RNA fraction of bone marrow-derived MDSCs, determined by RIP-qPCR.

**(G)** MDSCs were sorted from bone marrow-derived cells from WT and *Ythdf2*-cKO mice and were treated with actinomycin D. mRNA was collected at indicated time points after treatment and mRNA levels of *Adrb2*, *Metrnl* and *Smpdl3b* were measured by RT-qPCR. (n = 3 per group)

**(H)** BM-MDSCs were transduced with siRNAs targeting *Adrb2*, *Metrnl* and *Smpdl3b* simultaneously (3xKD). After 24-48 hr, the BM-MDSCs were purified and subjected to qPCR analysis of these three genes. (n = 3 per group)

**(I)** (left) WT, *Adrb2*, *Metrnl* and *Smpdl3b* knockdown MDSCs were co-cultured with LPS for 5 min. Immunoblot analysis of signaling associated proteins (as indicated) and phosphorylated (p-) proteins in these three type cells. (right) WT, 3xKD BM-MDSCs and BAY 11-7082 treated 3xKD BM-MDSCs (for 24hr) were co-cultured with LPS for 5 min. Immunoblot analysis of nuclear RELA.

**(J)** Bone marrow cells from CD45.1 mice were used to generate 3xKD BM-MDSCs using siRNA. MC38 tumor bearing *Ccr2*-knockout mice (CD45.2) were adoptively transferred with 1 x $10^6$ WT or 3xKD BM-MDSCs via i.v. injection. On the same day, mice were treated with local IR (20 Gy, one

dose). Three days after IR, the number of tumor-infiltrating $CD45.1^{+}CD11b^{+}Ly6C^{hi}$ $Ly6G^{-}$ cells was determined by flow. (n = 6 per group)

**(K)** Different BM-MDSCs as indicated were used for the transwell assay. The attached cells on the transwell membrane were visualized under a light microscope and quantified. (n = 3 per group)

**(L)** WT, 3xKD, *Ythdf2*-cKO and 3xKD-*Ythdf2*-cKO BM-MDSCs were used for adoptive transfer into MC38 tumor bearing *Ccr2*-knockout mice. On the same day, mice were treated with tumor-local IR (20 Gy, one dose). Three days after IR, the number of tumor-infiltrating $Ccr2^{+}CD11b^{+}Ly6C^{hi}$ cells was determined by flow. (n = 4 per group)

**(M)** Heatmap showing the qPCR analysis of relative *Cxcl16*, *Ccl5*, *Ccl2*, *Ccr7*, and *Il10* mRNA expression in WT, 3xKD, BAY 11-7082-treated WT and BAY 11-7082-treated 3xKD BM-MDSCs. The qPCR data were normalized to *Gapdh*. (n = 3 per group)

Data are represented as mean ± s.e.m., n, number of mice. One of two or three representative experiments was shown (E-M). Statistical analysis was performed using one-way ANOVA with Bonferroni's multiple comparison tests (E, K, L) or two-sided unpaired Student's *t*-test (F, G, H, J). $*P < 0.05$, $**P < 0.01$, $***P < 0.001$, and $****P < 0.0001$.

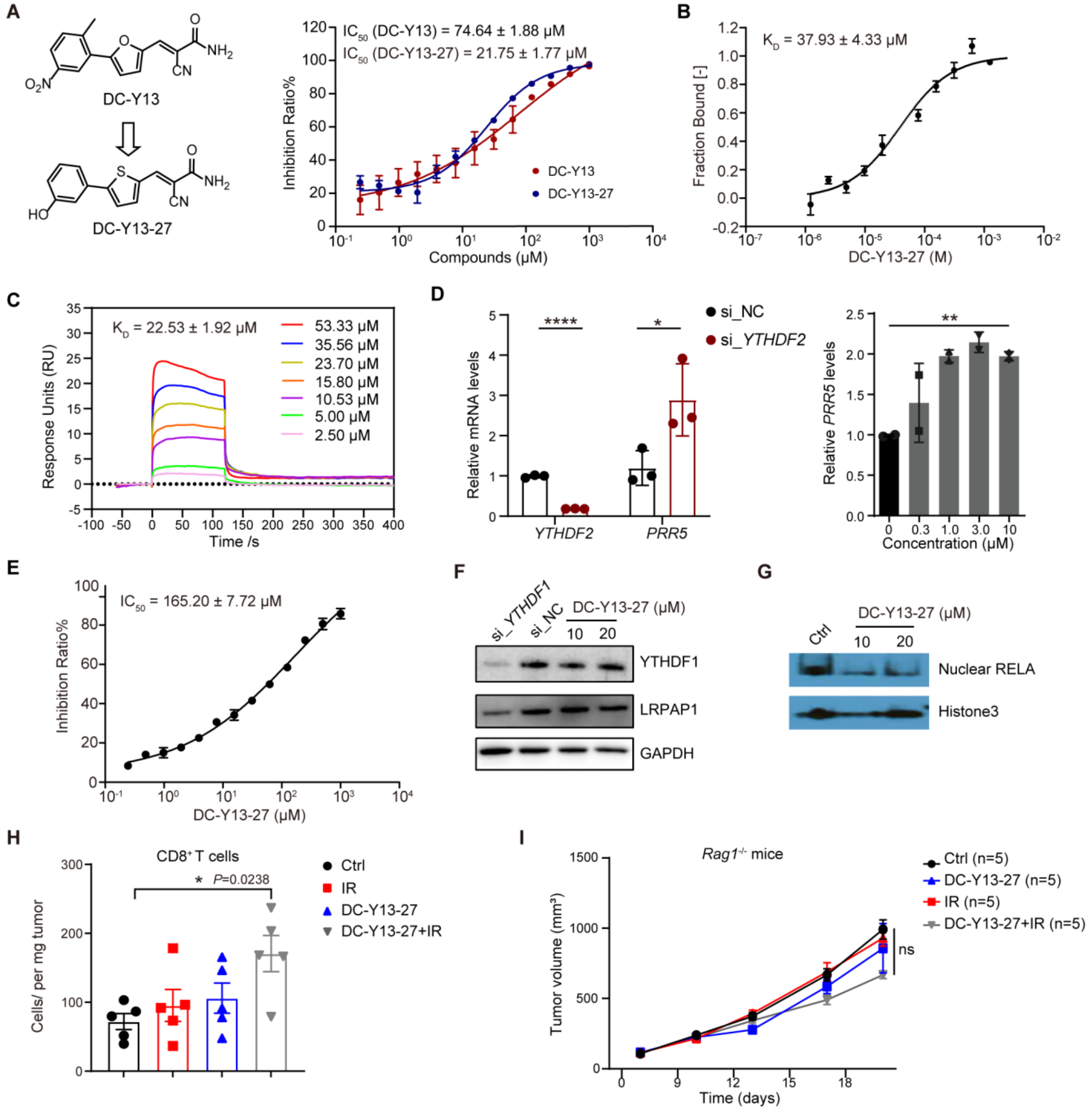


**Figure S6. The binding affinity, and selectivity of compound DC-Y13-27. Related to Figure 6.**

**(A)** The chemical structures of the compound DC-Y13 and DC-Y13-27 (left). Inhibitory activities ($IC_{50}$) of DC-Y13 (red) and DC-Y13-27 (blue) against YTHDF2 binding to $m^6A$ determined by AlphaScreen assay (right). Data are presented as mean ± SD.

**(B)** The MST binding curve of DC-Y13-27 and YTHDF2. Data are presented as mean ± SD.

**(C)** SPR binding curves of DC-Y13-27 and YTHDF2. The concentrations of the different compounds injected over the CM5 chip are indicated. Data are represented as mean ± s.e.m., one of three independent experiments is shown.

**(D)** (left) Hela cells were transduced with siRNA targeting *YTHDF2* and subjected to qPCR analysis of *YTHDF2* and its target gene *PRR5*. (right) Hela cells were treated with DC-Y13-27 in different concentration as indicated and subjected to qPCR analysis of *PRR5* (a target gene of YTHDF2 in Hela cells). Data are presented as the mean ± SD.

**(E)** Inhibitory activity ($IC_{50}$) of DC-Y13-27 against YTHDF1 binding to $m^6A$ detected via AlphaScreen assay. Data are presented as the mean ± SD.

**(F)** Immunoblot analysis of Hela cells treated with siRNA targeting *YTHDF1* or DC-Y13-27 to detect the protein levels of YTHDF1 and its target LRPAP1.

**(G)** Immunoblot analysis of nuclear RELA in BM-MDSCs treated with DC-Y13-27 (for 24 hr) in different dose as indicated.

**(H)** The numbers of tumor-infiltrating $CD8^+$ T cells in MC38 tumor-bearing mice with treatments as indicated. (Seven days after IR, n = 5 per group). Data are represented as mean ± s.e.m.

**(I)** MC38 tumor growth in *Rag1* knockout mice with IR and/or DC-Y13-27 treatment. (n = 5 per group). Data are represented as mean ± s.e.m.

Statistical analysis was performed using two-sided unpaired Student's *t*-test (D), one-way ANOVA with Bonferroni's multiple comparison tests (H), or two-way ANOVA test with corrections for multiple variables (I). $*P < 0.05$.
